# Novel Synthesis of C-Methylated Phytocannabinoids Bearing Anti-inflammatory Properties

**DOI:** 10.1021/acs.jmedchem.2c01988

**Published:** 2023-04-14

**Authors:** Yarden Lavi, Natalya M. Kogan, Louise M. Topping, Caojie Liu, Fiona E. McCann, Richard O. Williams, Aviva Breuer, Zhanna Yekhtin, Aviva Friedman Ezra, Ruth Gallily, Marc Feldmann, Raphael Mechoulam

**Affiliations:** † Medicinal Chemistry, Institute of Drug Research, The Hebrew University of Jerusalem, Jerusalem 91120, Israel; ‡ Institute of Personalized and Translational Medicine, Molecular Biology, Ariel University, Ariel 4070000, Israel; § Kennedy Institute of Rheumatology, University of Oxford, Oxford OX3 7FY, U.K.; ∥ 180 Life Sciences, Menlo Park, California 94025, United States; ⊥ Lautenberg Center of Immunology and Cancer Research, The Hebrew University of Jerusalem, Jerusalem 91120, Israel

## Abstract

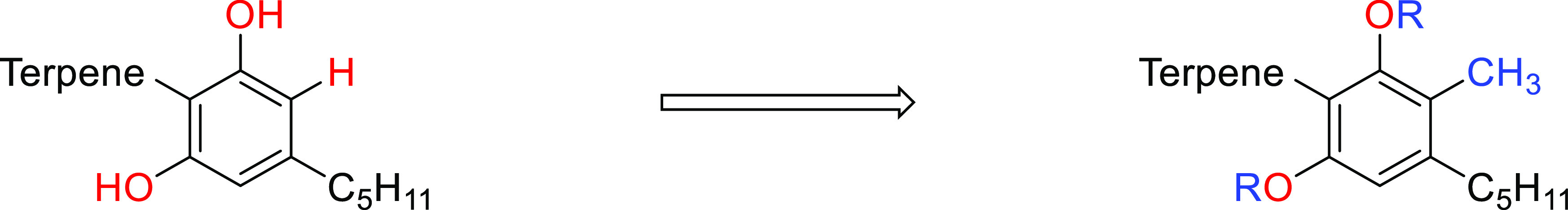

There is growing interest in non-psychoactive phytocannabinoids, namely cannabidiol (CBD), cannabigerol (CBG), and cannabichromene, as potential leads for novel therapeutic agents. In this study, we report on the development of new derivatives in which we methylated either position 4 of olivetol or the phenolic positions of olivetol, or both. We introduce a refinement on previously reported chemical procedures for phytocannabinoid derivatization as well as the biological evaluation of all derivatives in anti-inflammatory in vivo models. Compounds such as the CBD derivative, **2** and the CBG derivative, **11**, significantly reduced cytokine levels when compared to their parent compounds. Moreover, both of these derivatives proved to be as potent as dexamethasone for the inhibition of IL-1β. We believe that these new derivatives, as described herein, can be further developed as novel drug candidates for inflammatory conditions.

## Introduction

**Figure 1:**
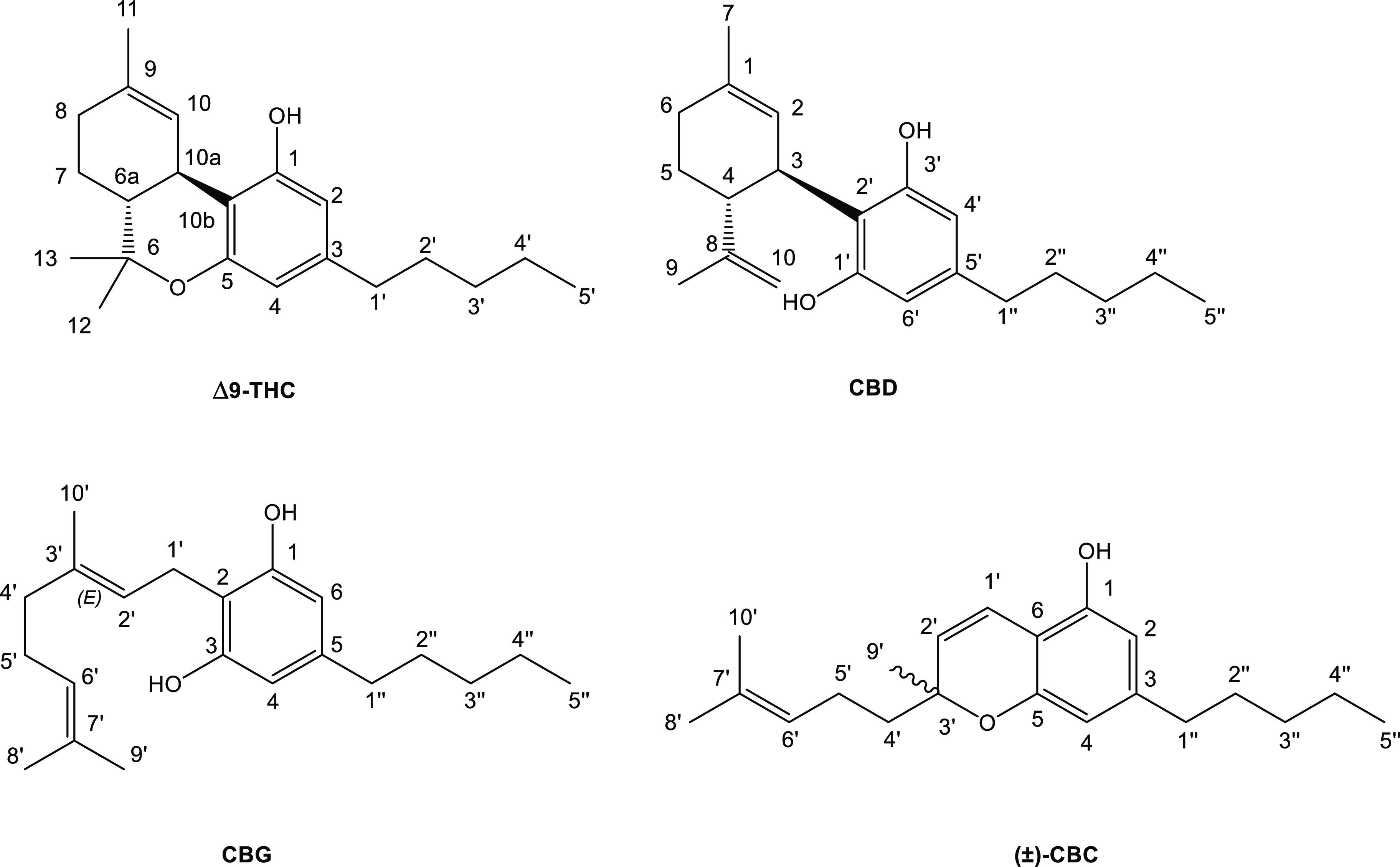
Four main phytocannabinoids and their numbering system.

Interest in the cannabis plant’s active compounds started in the 19th century but did not produce significant results until the mid-20th century when better analytical and synthetic tools were developed.^1^ Some of the compounds were successfully isolated in the 1930s and 1940s, but their correct characterization was only achieved by the 1960s as described in the review by Thakur et al.^2^ They were the first to isolate the psychotropic compound from cannabis in a pure form and therefore able to determine its structure and configuration.^3^ The compound was named Δ^9^-tetrahydrocannabinol (THC).^3−5^ Later, the same group isolated and characterized several other phytocannabinoids which did not have THC-like psychoactive properties: cannabidiol (CBD), cannabigerol (CBG), cannabichromene (CBC), etc.^6^ The structures of these phytocannabinoids are presented in Figure 1.

CBD, the major non-psychoactive component of cannabis, has also received significant interest, albeit secondary to THC, in the first two decades that followed its initial discovery. CBD’s chemistry and pharmacokinetics were established during the 1970’s and the 1980’s.^7^ The pharmacology of CBD differs from that of THC, and its biological targets are still being investigated.^8^ CBD is now known to possess anti-convulsive, sedative, anti-anxiety, anti-psychotic, anti-inflammatory, and other medicinal properties.^8^

CBG was discovered by Gaoni and Mechoulam and was considered a missing link in the biosynthesis of THC.^9^ However, its pharmacology was somewhat neglected.^10^ In recent years, CBG has been shown to possess anti-inflammatory properties, and some derivatives of CBG were synthesized and later tested in both animal models and human patients.^10−14^

CBC was isolated from hashish by Gaoni and Mechoulam soon after the discovery of the other aforementioned phytocannabinoids.^15^ They established that it caused ataxia and sedation, and later it was also determined to elicit some anti-inflammatory effects.^16,17^

The immune and inflammatory systems have evolved to serve as protectors against foreign organisms and pathogens that may cause damage and affect the healthy function of the body. When dysregulated, an attenuated or overactive immune response may lead to various pathological conditions, including the development of many cancers and autoimmune diseases such as rheumatoid arthritis, multiple sclerosis, inflammatory bowel disease, systemic lupus erythematosus, etc. Western medicine has introduced an array of anti-inflammatory drugs, with corticosteroids and non-steroidal anti-inflammatory drugs among the most common. These pharmaceuticals have long been known for their negative side effects, limiting their dosage and chronic use.^18^

Over the past two decades, there has been a growing interest in the non-psychoactive phytocannabinoids because they are deemed to be safe and well tolerated over periods of prolonged consumption while offering an array of health benefits. CBD primarily caught the attention of the scientific community, and many publications described it as anti-inflammatory, antioxidant, neuroprotective, etc.^19^ There have been many attempts to manipulate the structure of CBD in order to better understand its association with the observed biological activities and to introduce novel pharmaceutical derivatives that harness these benefits and further enhance the potency.^20,21^ Other phytocannabinoids such as CBG and CBC have received less attention in this regard, but in recent years the interest in their pharmacological potential has grown.^10−14,17,22−25^

Synthetic derivatization of the phytocannabinoids has focused mainly on the pentyl side-chain on the olivetolic moiety of these compounds.^2,26^ Substitutions on other positions of the olivetolic ring are less reported, and pharmacological evaluation of such derivatives is uncommon.^27−29^

Our group has demonstrated on quinone derivatives of CBD that methylation at position 4′ of the olivetol has a critical effect on the anti-cancer activity of these compounds.^30^ These findings led us to suspect that such derivatization might also affect the biological activity of other phytocannabinoids. Here. we report the synthesis of new methyl-substituted CBD, CBG, and CBC derivatives and show them to possess anti-inflammatory and pain-resolving properties in preclinical models. In addition, we report a novel method to synthesize C-methylated olivetol, which has been utilized for the synthesis of these new derivatives described herein. O-Methylated derivatives are also discussed.

## Results and Discussion

### Chemistry

#### C-Methylation of Olivetol

**Scheme 1:**
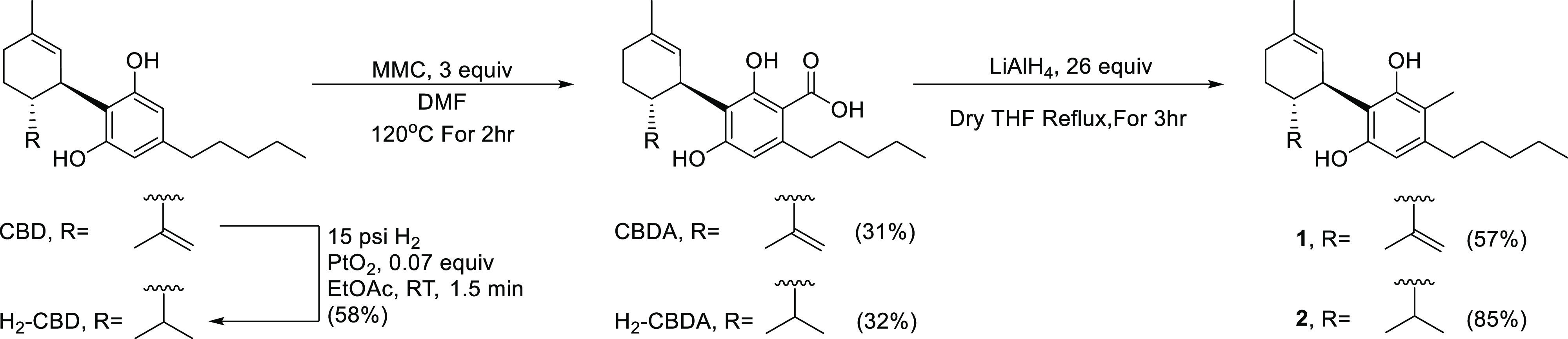
Method 1 for C-Methylation of Phytocannabinoids

Our group has previously published a method to C-methylate phytocannabinoids ortho to one of the phenols and the pentyl side-chain, which in the context of this work is referred to as method 1 (Scheme 1).^31,32^ In the first step, 3 equiv of methyl magnesium carbonate (MMC) in hot dimethylformamide (DMF) are used to carboxylate the cannabinoid ortho to one of the open phenols, which in the case of CBD yields cannabidiolic acid (CBDA, 31% yield). Then, the carboxylic acid is reduced with 26 equiv of lithium aluminum hydride (LiAlH_4_) in tetrahydrofuran (THF) under reflux to achieve an aliphatic carbon, which in the case of CBDA yields the compound named herein compound **1** in 57% yield. By employing method 1, **1** was obtained from CBD in 18% overall yield. **1** has been previously reported by our group as a precursor for a THC derivative but has not been evaluated for biological activity.^31^

CBD can be partially hydrogenated to form H_2_-CBD (Scheme 1).^33^ By employing method 1 to H_2_-CBD, we prepared the novel compound **2** in 27% overall yield.

Method 1 has unfortunately shown several disadvantages. First, the reaction with MMC resulted in low yields, never exceeding 35%. Moreover, the reaction did not go to completion, and the resultant crude mixture contained a great number of impurities from side reactions. The crude product required repetitive chromatographic separations to yield a sufficiently pure product to continue the synthesis. Second, the reduction step required a high excess of the pyrophoric hydride and a reflux to yield the methylated product and not the alcohol, which made this step a relatively dangerous one. Overall, the process proved to be both expensive and long since it required expensive reagents and numerous purification steps to achieve a relatively low yield of the final product.

We have therefore opted to search for an alternative method to achieve C-methylated cannabinoids with a better yield and an easier purification process that requires cheaper reagents. We decided that a more versatile approach would be to prepare a methylated olivetol to which we can later add the terpene of choice.

**Scheme 2:**
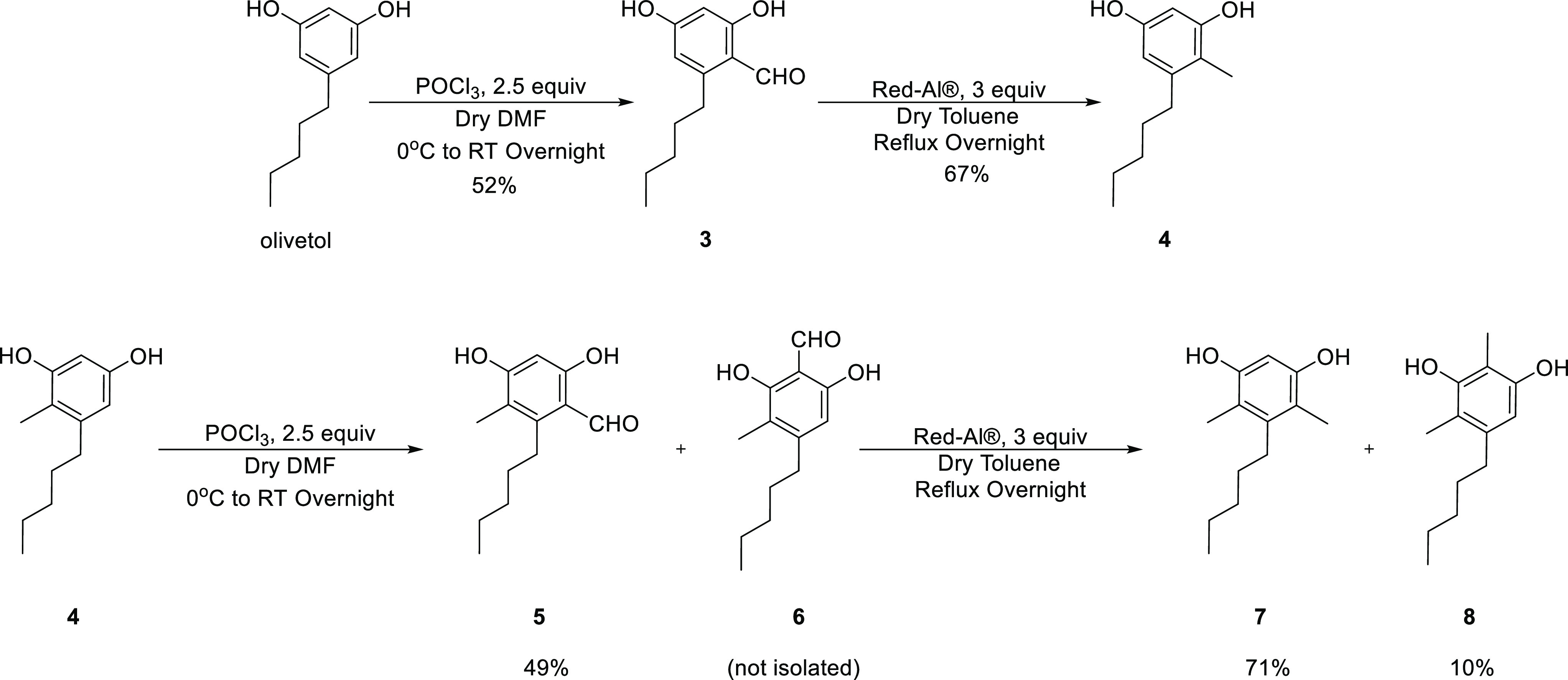
C-Methylation of Olivetol to Compound **42** and C-Methylation of Compound **4** to Compounds **7** and **8**

Monomethyl olivetol has been synthesized before, but the process had numerous steps and was therefore impractical if the goal was a building block for further development.^34^ The method we developed, which is named method 2 throughout this paper, consists of two steps (Scheme 2). First, the starting material, olivetol, was formylated by 2.5 equiv of phosphoryl chloride in DMF to obtain aldehyde **3** in 52% yield.^35^ This reaction had the benefits of being regioselective to the position ortho to the phenol and the pentyl with no side reactions. Any unreacted olivetol is recoverable from this reaction, and there is no loss of starting material, which can be used again to raise the overall yield. Second, aldehyde **3** is reacted with a safer alternative of LiAlH_4_, namely lithium bis(2-methoxyethoxy)aluminum hydride (Red-Al), in refluxing toluene to obtain the desired methylated olivetol derivative **4** in 67% yield.^36^ The number of equiv needed to achieve the methylated product from the aldehyde is considerably smaller, namely 3 equiv, which makes this reaction safer and more efficient than its counterpart from method 1. As was the case in the first step, the unreacted aldehyde can be easily recovered from the reaction mixture and used again. Method 2 can be easily repeated for **4** to obtain aldehyde **5** and eventually the dimethylated olivetols **7** and **8** in 71 and 10% yields, respectively. When method 2 was applied to **4,** a small amount of byproduct in which the formylation took place between the two phenols was observed (herein named compound **6**). This byproduct **6** could not be separated at that stage; its reduction with Red-Al led to **8**.

#### C-Methylated CBD Derivatives

**Scheme 3:**
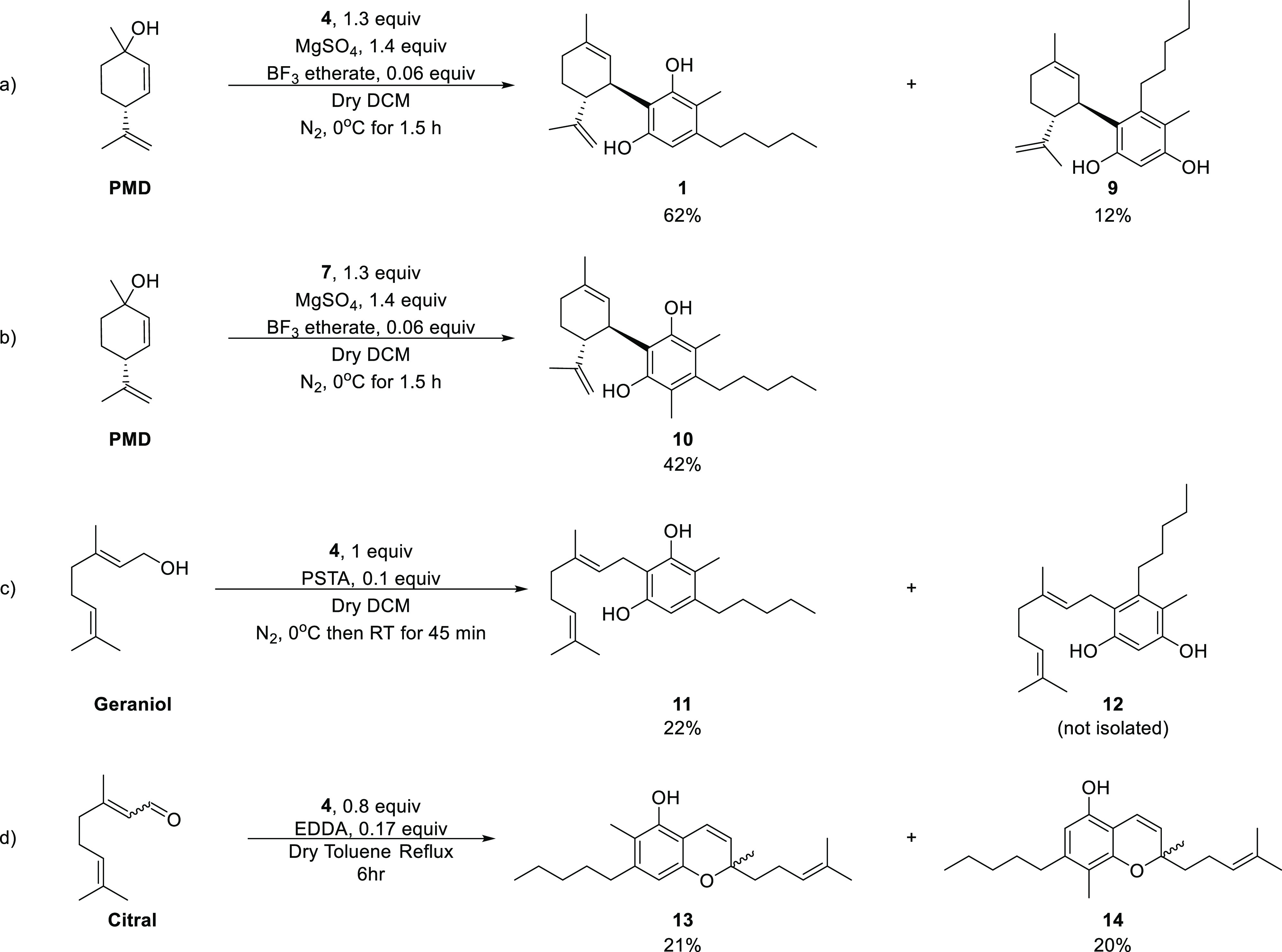
Synthesis of C-Methylated Phytocannabinoids from Compounds **4** and **7**

Reaction of 1.3 equiv of **4** and (1*S*,4*R*)-*p*-mentha-2,8-dien-1-ol (PMD) in the presence of catalytic boron trifluoride diethyl etherate in cold methylene chloride (DCM) resulted in **1** (62% yield) and **9** (12% yield, Scheme 3a).^37^**1** resembles CBD’s structure, and **9** is its structural isomer which does not have a known naturally occurring equivalent. When **7** was reacted with PMD in the same manner as **4**, the dimethylated compound **10** was obtained in 42% yield (Scheme 3b).

#### C-Methylated CBG Derivatives

The C-methylated CBG, **11**, was synthesized from an equimolar mixture of **4** and geraniol in the presence of catalytic *p*-toluenesulfonic acid (PTSA) in cold DCM (Scheme 3c).^38^ It is important to mention that, as in the case of **1** and **9**, this reaction yielded both **11** and a **9**-like isomer (named herein compound **12**). However, the acid present in the reaction causes a minimal isomerization of a geranyl double bond and leads to an impurity of both **11** and **12**. We were able to purify **11** by recrystallization from pentane. Unfortunately, this method was not helpful in the case of **12,** which could not be purified by chromatography to more than 85% purity. Hence, compound **12** was not biologically evaluated in this work. Compound **11** has been previously synthesized by a biosynthetic method. However, to the best of our knowledge, it was not assessed for its pharmacological activity.^39^

#### C-Methylated CBC Derivatives

Compound **4** was reacted with 1.25 equiv of citral in the presence of catalytic ethylenediamine diacetate (EDDA) in refluxing toluene, which resulted in **13** and **14** in 21 and 20% yields, respectively (Scheme 3d).^40^ The two isomers could be separated by chromatography and their assigned structures were deduced by NMR spectroscopy (see Supporting Information).

#### O-Methylated CBD and CBG Derivatives

**Scheme 4:**
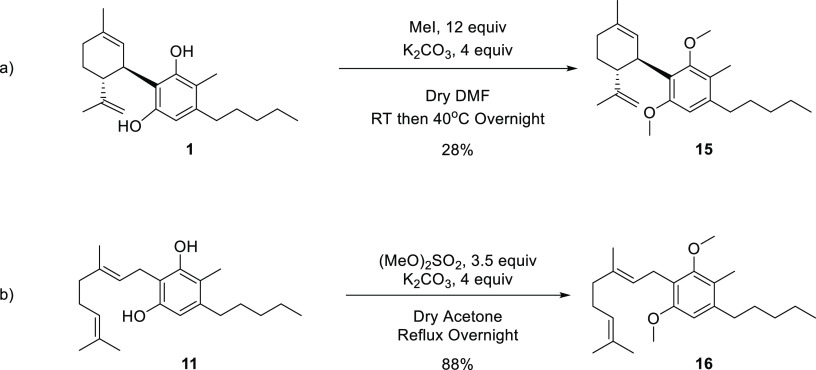
O-Methylated Derivatives of CBD and CBG

This derivatization aimed to evaluate the necessity of open phenols to the activity of **1** and **11**. We therefore prepared compound **15** from **1** using 12 equiv of iodomethane in DMF in the presence of 4 equiv potassium carbonate (Scheme 4a).^41^ This reaction did not prove efficient as it resulted in a low yield of **15**, 28%, and required repetitive chromatographies. Hence, this reaction was modified in the derivatization of **11**. We used the much more potent methylating agent, dimethyl sulfate, using 3.5 equiv of it in the presence of 4 equiv of potassium carbonate in refluxing acetone, to generate **16** from **11** in 88% yield (Scheme 4b).^34^

### Biological Evaluation

#### Anti-inflammatory Properties

**Figure 2:**
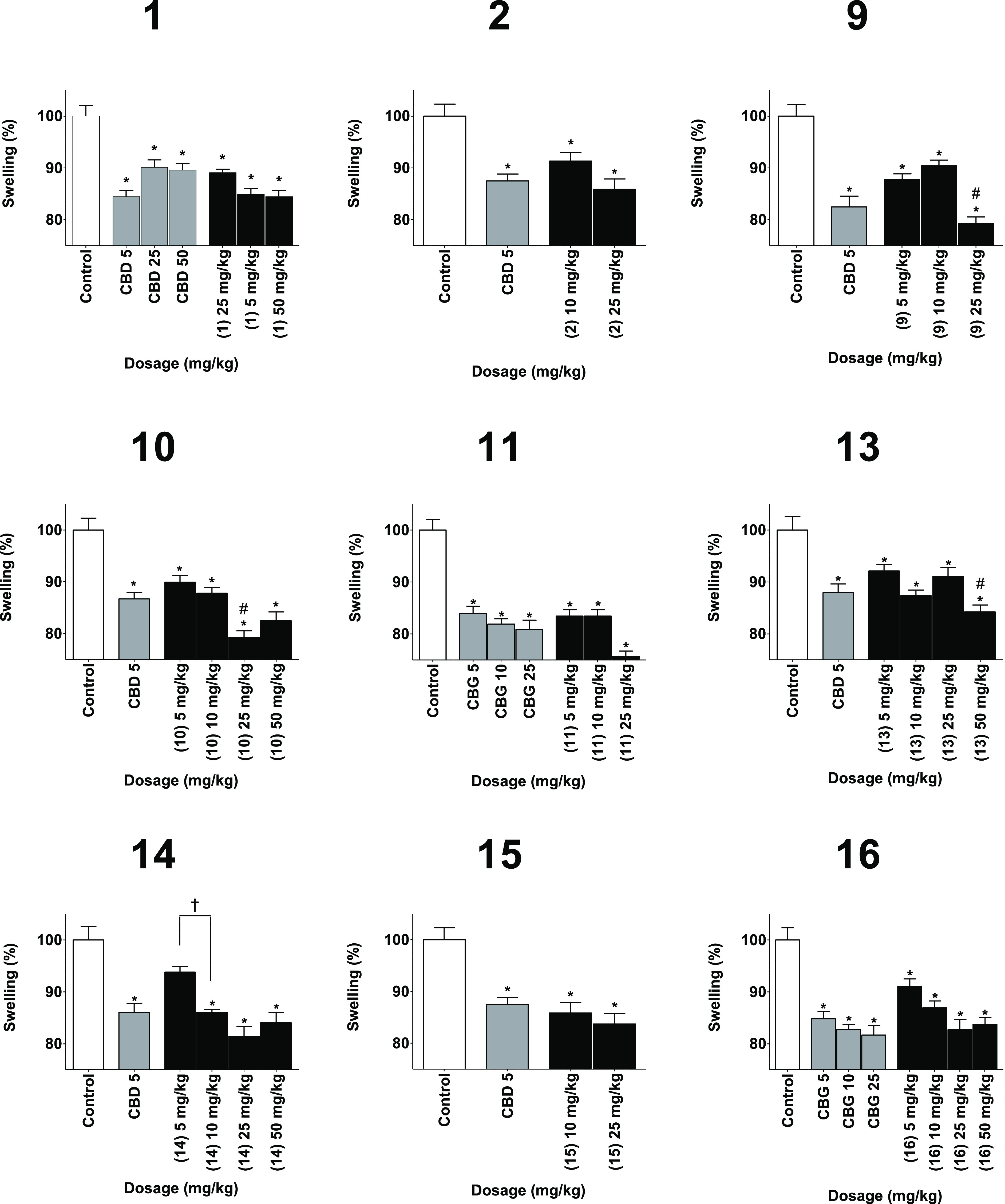
Reducing swelling capabilities of the phytocannabinoids (black) compared to the vehicle as a negative control (white) and the appropriate phytocannabinoids as the positive control (gray). Measurements were done 6 h after injection. Results are presented as column + SEM. Swelling reduction is presented as a percentage compared to the control group. Statistical comparison by one-way analysis of variance (ANOVA) (*P*-value < 0.0001) and post hoc analysis by Tukey’s test. * *P*-value < 0.05 compared to control. # *P*-value < 0.05 compared to the positive control. † *P*-value < 0.05 in the presented comparison.

**Figure 3:**
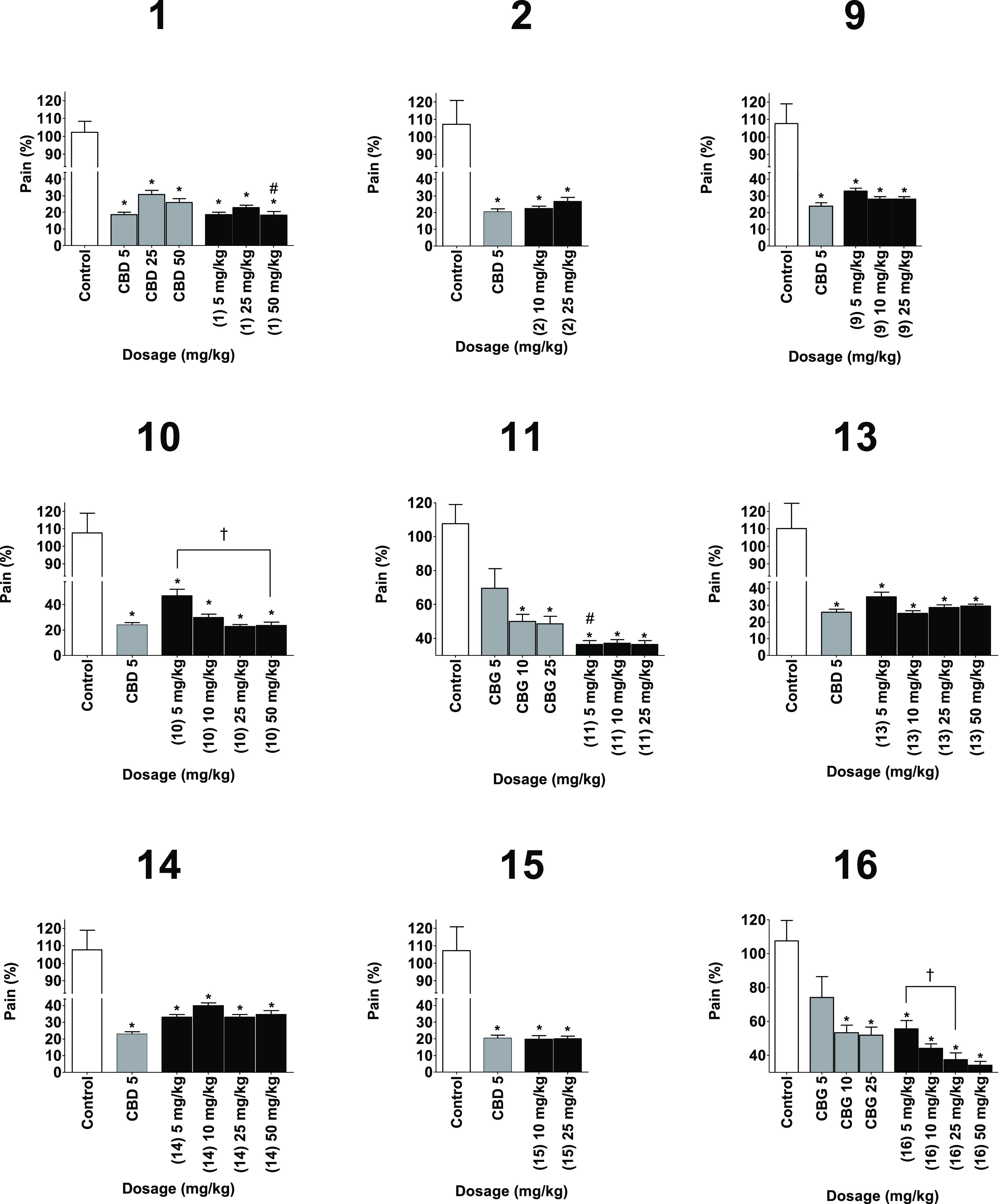
Reducing pain sensation capabilities of the phytocannabinoids (black) compared to vehicle as the negative control (white) and the appropriate phytocannabinoids as the positive control (gray). Measurements were done 6 h after injection. Results are presented as column + SEM. Pain sensation reduction is presented as a percentage compared to the control group. Statistical comparison by one-way ANOVA (*P*-value < 0.0001) and post hoc analysis by Tukey’s test. * *P*-value < 0.05 compared to control. # *P*-value < 0.05 compared to the positive control. † *P*-value < 0.05 in the presented comparison.

**Figure 4:**
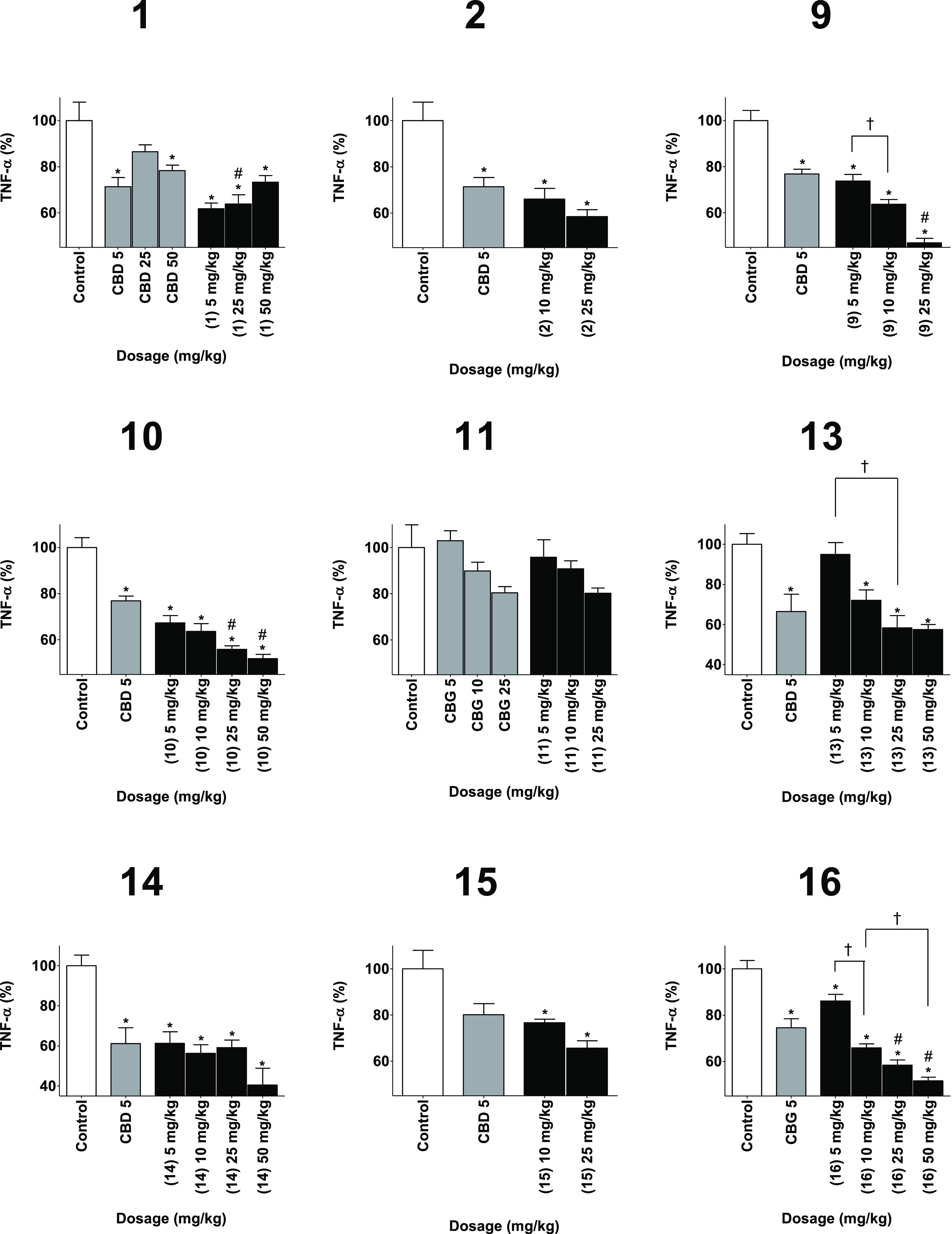
Reducing TNFα levels capabilities of the phytocannabinoids (black) compared to vehicle as the negative control (white) and the appropriate phytocannabinoids as the positive control (gray). TNFα was measured 24 h after injection. Results are presented as column + SEM. TNFα reduction is presented as a percentage compared to the control group. Statistical comparison by one-way ANOVA (*P*-value < 0.0001) and post hoc analysis by Tukey’s test. * *P*-value < 0.05 compared to control. # *P*-value < 0.05 compared to the positive control. † *P*-value < 0.05 in the presented comparison.

All the phytocannabinoid derivatives prepared were assessed for anti-inflammatory activity in three different in vivo assays of inflammation: paw swelling (Figure 2), pain sensation in the paw (Figure 3), and circulating tumor necrosis factor α (TNFα) (Figure 4). Derivatives of CBD were compared to CBD as a positive control and to vehicle as a negative control. Derivatives of CBG were compared to a vehicle control group and to CBG as a positive control. Derivatives of CBC were compared to a vehicle-negative control group and a CBD-positive control group. Inflammation was induced by local injection of zymosan to the paw, followed by injection of either test compound, positive control of negative control. Swelling and pain were measured 6 h after the injection, while TNFα was measured after 24 h.

All compounds presented a significant improvement of swelling and pain sensation compared to control. A dose of 25 mg/kg of **9** significantly reduced swelling compared to positive control. Similar observations were made for 25 mg/kg of **10** and 50 mg/kg of **13**. As for pain sensation, compound **1** showed a significant reduction at a dose of 50 mg/kg compared to a similar dose of CBD. This was also observed for compound **11** at a dose of 5 mg/kg.

In the case of TNFα, a significant reduction of its concentration was observed in all tested compounds but **11**. A significant improvement compared to positive control was observed for 25 mg/kg of **1**, **9**, **10,** and **16**.

For some of the parameters of the biological evaluation, compounds **9** and **10** present a linear dose–response curve. **9** presented a linear decrease in circulating TNFα in increased doses, and **10** presented a decrease in pain sensation in increased doses. CBD has previously been shown in this biological model and others to have a bell-shaped dose–response behavior.^42^

Both **13** and **14** showed an anti-inflammatory activity which was comparable to CBD. **13** showed a linear dose response in the TNFα assay but not in the pain and swelling assays. **14** showed a linear dose–response in the swelling assay but not in the pain and TNFα assay. We therefore concluded that methylation either at position 2 or 4 of CBC does not reduce the compound’s anti-inflammatory activity.

The biological evaluation of **11** and **16** revealed that mono-methylation of CBG and its methoxylation to **11** and **16,** respectively, led to a partial improvement of anti-inflammatory activity in our assays. **11** showed an improved pain tolerability at 5 mg/kg compared to CBG, and **16** showed similar improvement at 25 mg/kg in this test. Moreover, when comparing the dose–response behavior of **11** and **16**, they present a linear behavior in some assays but not in all of them.

#### Reducing Circulating Cytokine Levels

**Figure 5:**
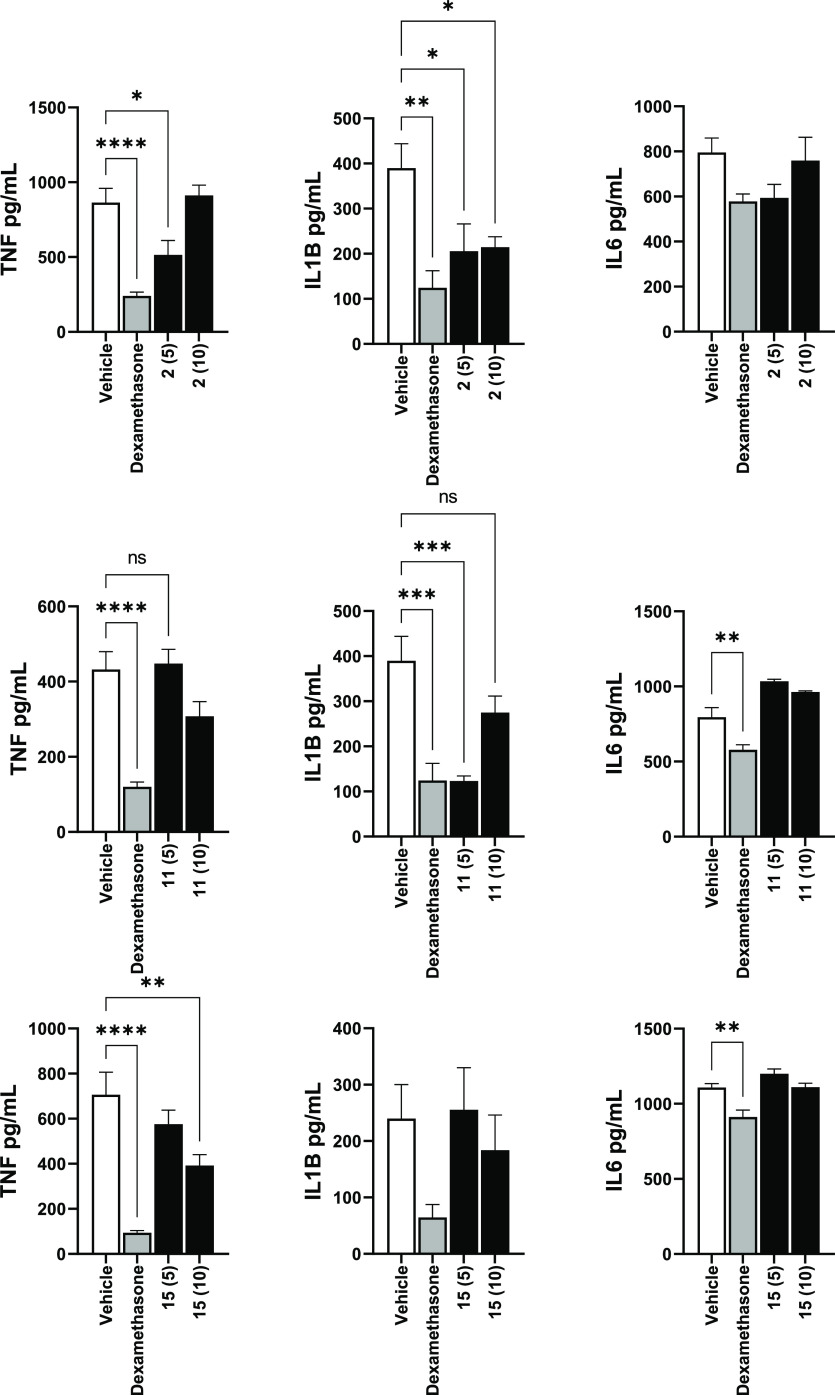
CBD derivatives **2** and **15** and CBG derivative **11** significantly reduce plasma TNFα in mouse LPS-induced peritonitis. The results are presented as columns + SEM. Statistical analysis by one-way ANOVA, compared to vehicle, with Dunnett’s multiple comparison test. * *P* < 0.05, ** *P* < 0.01, *** *P* < 0.001, and **** *P* < 0.0001.

To further confirm the anti-inflammatory effects of these derivatives, we decided to test the ability of a few of them to reduce circulating blood cytokines such as TNFα, interleukin 1-β (IL-1β), and interleukin 6 (IL-6) induced by a single injection of lipopolysaccharides (LPS) into the peritoneal cavity in a mouse peritonitis model. Out of the nine compounds presented in this work, we selected **2**, **11,** and **15** since they better structurally resemble their parent phytocannabinoid and thus have the least potential to produce toxic side effects. The corticosteroid dexamethasone, an established anti-inflammatory drug, was used as a positive control (Figure 5). At 5 mg/kg, given 1 h before LPS, **2** significantly reduced plasma TNFα and IL-1β. However, the levels of IL-6 were not significantly affected.

We also compared **2** with its parent compound, CBD using an assay that involves activation of the inflammasome. The results show that **2** inhibits IL-1β expression effectively at a concentration of 5 μM and is significantly more potent than CBD.

**Figure 6:**
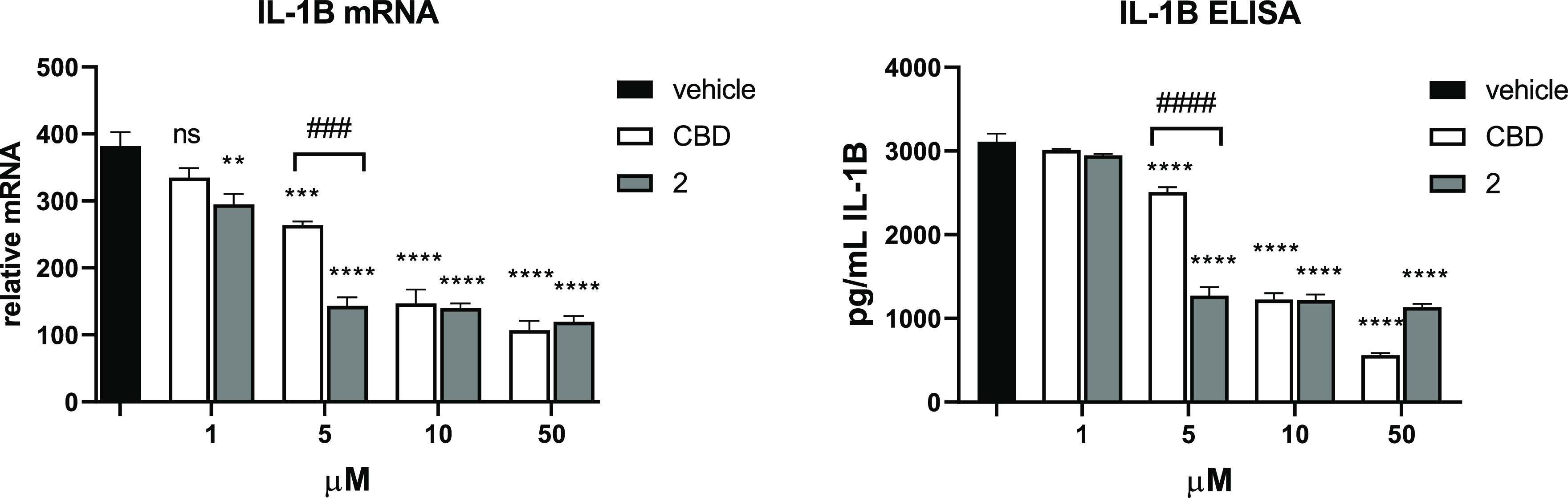
**2** is more potent than CBD in inhibiting the expression of IL-1β in human macrophages. Measurement of IL-1β messenger RNA by qRT-PCR (left) and protein by enzyme-linked immunosorbent assay (ELISA) (right) in LPS-primed and adenosine 5′-triphosphate (ATP)-activated human macrophages treated with CBD or **2** (*n* = 3). Bars represent means ± SD. ** *P* < 0.01, *** *P* < 0.001, and **** *P* < 0.0001 versus cells treated with vehicle alone (one-way ANOVA); ### *P* < 0.001 CBD versus **2** treatment (Tukey’s multiple comparison test).

At 5 mg/kg, administered intraperitoneally 1 h before LPS, **11** significantly reduced plasma IL-1β; however, the levels of TNFα and IL-6 were not significantly affected (Figure 6). Blood was collected for subsequent analysis 90 min after LPS administration.

At 10 mg/kg, **15** significantly reduced plasma TNFα; however, levels of IL-1β and IL-6 were not significantly affected. Blood was collected and plasma isolated and frozen for subsequent analysis 90 min after LPS administration.

## Conclusions

The objective of the presented study was to investigate how two types of previously unreported modifications of the structures of phytocannabinoids alter their anti-inflammatory activity. A total of nine novel compounds were synthesized by derivatization of the resorcinol moiety of phytocannabinoids. We evaluated two structural changes: an alkylation at position 4 of the resorcinol ring and methoxylation of the phenols. These two types of derivatives were aimed to improve the anti-inflammatory effect compared to the parent phytocannabinoids. In this work, we were able to improve upon synthetic methods previously widely used and to prepare a wide array of new derivatives.

Given the uncertainty surrounding the receptors through which CBD, CBG, and CBC act, we focused on measuring the effects of the derivatives on inflammatory read-outs rather than their affinity for different receptors. We plan to elaborate on the mechanism(s) of action of these new compounds in future work.

Our observations of the CBD derivatives showed that O- or C-methylation preserves, and in some cases even improves the compounds’ efficacy. **2** and **15** significantly reduce cytokine levels. Moreover, **2** is a more potent inhibitor of IL-1β expression than CBD, and as active as the approved glucocorticoid, dexamethasone for IL-1β inhibition.

From the presented results for the modifications of CBG, we conclude that O-methylation and C-methylation may provide an improvement to the compound’s anti-inflammatory activity. **11** proved to significantly reduce cytokine levels, exceeded CBG’s potency in some of our assays and exhibited a comparable activity to dexamethasone in the inhibition of IL-1β. **16** also demonstrated an improved biological activity compared to CBG in some of the assays but not in all of them. Hence, we decided to halt further development of new methoxylated derivatives and focus our attention on C-methylation in the presented work.

In the case of CBC derivatives, we conclude from our observations that C-methylation in either position 2 or 4 of CBC preserved its biological activity while showing an altered dose–response profile. Further investigation is needed to fully understand CBC’s SAR as a potential anti-inflammatory phytocannabinoid.

In conclusion, we developed nine new derivatives of the known CBD, CBG, and CBC phytocannabinoids, focusing on the C-methylation of the fourth position of the olivetolic moiety of these compounds. Our observations indicate that for most of these derivatives, the anti-inflammatory activity is retained, while some exhibit an improved biological response. **2** was found to be the most preferential for further development, as it was effective in both TNFα and IL6 assays (unlike **9** that was effective only in TNFα assay). **11** was effective in both of these assays, but as has been previously described, it was a less applicable drug candidate.

We believe that the described derivatives, most notably **2,** have the potential to be further developed as novel drug candidates for use in inflammatory conditions.

## Experimental Section

### Materials and General Methods

#### Chemistry

All the chemicals and solvents used were purchased from established commercial sources and used without any further purification procedures.

Newly synthesized cannabinoids and intermediate compounds were characterized by ^1^H NMR, ^13^C NMR, and either gas chromatography–mass spectrometry (GCMS) or liquid chromatography–electrospray ionization mass spectrometry (LC–ESI-MS). The melting point was determined for the solid compounds. For some final compounds sent for biological evaluation, analytical amounts were purified by preparative high-performance liquid chromatography, courtesy of Prof. Dan Gibson’s group.^44^ Compounds with asymmetric centers were also measured for optical rotation. Carboxylic acid intermediates were analyzed by LC–ESI-MS and not GCMS since they underwent decarboxylation at high temperatures.

All phytocannabinoid derivatives sent for biological evaluation were >95% pure according to their GCMS.

GCMS analysis was done with a HP Hewlett Packard GCD G1800B system equipped with a silica column (Agilent Tech. 122-5532 DB-5MS 30 m × 0.25 mm, 0.25 μm). Experimental conditions were: inlet, 250 °C; detector, 280 °C; splitless injection time; initial temperature, 90 °C; initial time, 3.00 min; rate, 25 °C/min; final temperature, 280 °C; helium flow rate, 1.0 mL/min. The software used was GCD Plus ChemStation.

NMR data were collected on either a Bruker AVANCE IIITM HD 500 MHz spectrometer or a Varian VXR-300S spectrophotometer. The data were processed using MestReNova-10.0.2 or Varian’s server program for experiment execution and analysis. All chemical shifts are reported in ppm. ^1^H and ^13^C NMR chemical shifts were referenced with the individual solvent residual peaks of the respective NMR solvents used.

Specific rotations were measured with a PerkinElmer 141 polarimeter in a 2.00 dm cell.

##### Cannabidiolic Acid

6.28 g of CBD (20 mmol) were dissolved in 30 mL of a 2 M MMC solution in DMF (60 mmol). A condenser was attached, and the reaction was heated to 120 °C for 2 h, monitoring by thin-layer chromatography (TLC). The reaction was worked up by pouring it into an ice-cold 10% w/v HCl solution. The aqueous phase was extracted three times with ether. The combined organic phase was then dried over MgSO_4_ and evaporated to give a dark purple syrupy crude. The CBDA was purified by silica gel column chromatography using EtOAc/MeOH/AcOH (10%:2%:1%, respectively) in hexanes. CBDA was obtained as yellow foam. Yield: 2.3 g (31%). Compound’s characteristics were in accordance with previously published literature.^43^

##### H_2_CBD

5 g (15.92 mmol) of CBD and 250 mg (1.1 mmol, 5% w/w) of platinum oxide were added to a pressure resistance flask and dissolved in 10 mL of EtOAc. The reaction flask was then vacuumed and pressurized with hydrogen gas to 15 psi. The reaction flask was placed on an automatic shaker, and the pressure was maintained for 1.5 min. The hydrogen was then removed by vacuum. The mixture was filtered and evaporated. The crude product was purified by silica gel column chromatography (TLC 10% EtOAc/hexanes). Yield: 2.9 g (58%). Analytical properties were in accordance with previously published literature.^33^

##### H_2_-CBDA

H_2_-CBDA was prepared from 3 g of H_2_-CBD by the same method as described for CBDA. Yield: 1.1 g (32%). ^1^H NMR (300 MHz, CDCl_3_): δ 11.89 (s, 1H), 6.75 (s, 1H), 6.29 (s, 1H), 5.51 (s, 1H), 4.07–3.93 (m, 1H), 3.03–2.74 (m, 2H), 2.26–2.02 (m, 2H), 1.86–1.81 (m, 1H), 1.79 (s, 3H), 1.68–1.51 (m, 3H), 1.41–1.30 (m, 3H), 0.97–0.82 (m, 9H). ^13^C NMR (75 MHz, CDCl_3_): δ 176.73, 164.40, 161.47, 147.92, 124.65, 115.04, 112.44, 102.78, 54.17, 43.91, 36.71, 35.22, 32.19, 31.40, 30.68, 28.00, 23.84, 22.68, 22.17, 21.85, 16.71, 14.22. LCMS: ES (+): *m*/*z* 361 [M + H], ES (−): *m*/*z* 359 [M – H], *t*_R_: 15.78 min.

##### 4′-Methylcannabidiol (**1**)—Method 1

2.3 g (6.4 mmol) of CBDA were dissolved in a minimal amount of dry THF under nitrogen. The solution was then slowly added to a flask containing a pre-cooled suspension of 6.3 g (167 mmol) of LiAlH_4_ in dry THF (57 mL, cooled to −20 °C using an ice/acetone bath). A condenser was attached, and the reaction was heated to reflux for 3 h, monitoring by TLC. Upon full consumption of the starting material, the reaction was cooled to −20 °C for the workup. EtOAc was added dropwise to neutralize the remaining LiAlH_4_, followed by ethanol, methanol, and ice until there was no visible reaction of the LiAlH_4_ in the flask. The reaction suspension was then acidified with a 10% w/v HCl solution to pH 1. The aqueous phase was extracted three times with EtOAc, washed with sat. NaHCO_3_ to pH 8 and brine to neutral pH. The organic phase was dried over MgSO_4_ and evaporated. The crude product was purified by silica gel column chromatography (TLC 10% EtOAc/hexanes). The compound was code-named HUM-216; was obtained as a yellow syrup. Yield: 1.2 g (57%). ^1^H NMR (300 MHz, CDCl_3_, ppm): δ 6.16 (1H, s), 5.55 (1H, s), 4.66 (2H, m), 3.9 (1H, s), 2.43 (2H, t, *J* = 7.5 Hz), 2.2 (2H, m), 2.05 (3H, s), 1.78 (3H, s), 1.63 (3H, s), 1.5 (2H, t, *J* = 6 Hz), 1.3 (4H, m), 0.88 (3H, t, *J* = 6 Hz); ^13^C NMR (75 MHz, CDCl_3_, ppm): δ 140.72, 124.32, 110.84, 108.05, 65.94, 64.64, 45.98, 37.05, 33.54, 31.86, 30.38, 30.02, 28.45, 23.76, 22.62, 20.85, 20.20, 15.20, 14.10; GCMS: *m*/*z* 328, *t*_R_: 12.6 min. [α]_D_ in EtOH: −69.1°.

##### (**1**)—Method 2

1 g (5.16 mmol) of **4** was dissolved in 16.5 mL of dry DCM under nitrogen and was added via syringe to a nitrogen-flushed flask containing 0.68 g of MgSO_4_ (5.65 mmol). Then the flask was cooled to 0 °C, and 32 μL (0.26 mmol) of BF_3_ diethyl etherate were added. 0.63 g (4.12 mmol) of PMD were separately dissolved in 10.6 mL of dry DCM and cooled to 0 °C. The cold PMD solution was then added dropwise with high stir to the cold **4** and BF_3_ diethyl etherate solution. The reaction was then stirred at 0 °C for 1.5 h. The reaction was quenched by addition of sat. NaHCO_3_ and the layers separated by separatory funnel. The water phase was then extracted three times with DCM. The combined organic phase was washed with brine, dried over MgSO_4_ and evaporated. The crude product was purified by silica gel column chromatography (TLC 20% EtOAc/hexanes). Yield: 0.84 g (62%).

##### 4′-Methyl-10-dihydrocannabidiol (**2**)

4′-Methyl-10-dihydrocannabidiol (**2**) was prepared from 1.1 g of H_2_-CBDA by the same method described for **1** (method 1). Yield: 0.85 g (85%). The compound was code-named HUM-217; it was obtained as yellow syrup. ^1^H NMR (300 MHz, CDCl_3_, ppm): δ 6.28 (s, 1H), 6.13 (s, 1H), 5.01 (s, 1H), 3.99–3.70 (m, 1H), 2.50 (t, *J* = 7.8 Hz, 2H), 2.10 (s, 4H), 1.79 (s, 3H), 1.77–1.59 (m, 2H), 1.58–1.44 (m, 2H), 1.42–1.31 (m, 5H), 0.94–0.82 (m, 9H). ^13^C NMR (75 MHz, CDCl_3_, ppm): δ 140.71, 140.22, 124.93, 77.52, 77.10, 76.67, 43.43, 35.78, 33.64, 32.01, 30.70, 30.13, 27.79, 23.73, 22.65, 22.61, 22.08, 21.77, 16.37, 14.13. GCMS: *m*/*z* 330 *t*_R_: 12.5 min. [α]_D_ in EtOH: −59.6°.

##### 2,4-Dihydroxy-6-pentylbenzaldehyde (**3**)

11.6 mL of phosphoryl chloride (0.125 mol) were slowly and dropwise dissolved in an ice-cold anhydrous DMF (33 mL) under a nitrogen atmosphere. To it was added dropwise a 24.7 mL solution of 9 g (0.05 mol) of olivetol in anhydrous DMF. The reaction was slowly warmed to room temperature and stirred overnight. The reaction was cooled on ice and to it was added dropwise 61.8 mL of ice-cold water. While still on ice, a 20% solution of sodium hydroxide was added to the reaction until pH of 10 was achieved. The reaction was then refluxed for 10 min. The reaction was acidified using conc. hydrochloric acid until pH of 1 was achieved. The aqueous reaction solution was then extracted four times with ethyl acetate. The organic extractions were washed with brine, dried over magnesium sulfate (MgSO_4_), and the solvent was evaporated on vacuum. The crude oil was dry loaded on silica gel and purified by column chromatography [TLC 20% ethyl acetate (EtOAc)/hexanes] to receive **3** as a pale-yellow solid. Yield: 5.5 g (52%). Product characteristics were in accordance with known literature.^35^

##### 1,3-Dihydroxy-4-methyl-5-pentylbenzene (**4**)

4.4 g of **3** (21.3 mmol) were dissolved in 42 mL of anhydrous toluene under a nitrogen atmosphere and cooled on an ice bath for 5 min. To the cold suspension was added 21 mL of a 60% w/w solution of Red-Al in toluene (63.9 mmol). The reaction was then refluxed overnight. The reaction was cooled on an ice bath, and 5 mL of brine were added dropwise to avoid spillage. The mixture was partitioned between ether and water, and the aqueous phase was acidified to pH of 1 using a 20% w/v sulfuric acid solution. The now acidic aqueous phase was extracted three times with ether. The combined extractions were washed with sat. NaHCO_3_ and brine and dried over MgSO_4_. The evaporated crude was purified by column chromatography (TLC 20% EtOAc/hexanes) to receive **4** as a white solid. Yield: 2.8 g (67%). This compound was previously reported as an oil.^34^ However, it is a solid at room temperature after complete evaporation on high vacuum. ^1^H NMR (300 MHz, CDCl_3_): δ 6.26 (d, *J* = 2.5 Hz, 1H), 6.20 (d, *J* = 2.5 Hz, 1H), 5.15–4.75 (m, 2H), 2.51 (t, *J* = 9.4, 8.0 Hz, 2H), 2.09 (s, 3H), 1.59–1.45 (m, 2H), 1.33 (dt, *J* = 7.2, 3.7 Hz, 4H), 0.90 (q, *J* = 9.1, 6.3 Hz, 3H). ^13^C NMR (75 MHz, CDCl_3_, ppm): δ 154.45, 153.38, 143.98, 114.50, 108.59, 100.44, 33.75, 31.84, 30.03, 22.57, 14.06, 10.57; mp: 60–61 °C. GCMS: *m*/*z* 194, *t*_R_: 9.66 min.

##### 2,4-Dihydroxy-5-methyl-6-pentylbenzaldehyde (**5**)

This compound was synthesized from 2 g (10.3 mmol) of **4** by the same method as described for **3**. Compound **5** was obtained as a yellow crystalline solid. Yield: 1.1 g (49%). Both GCMS and NMR indicated the presence of a 10% impurity. The secondary product of the reaction was an aldehyde addition between the two phenolic groups, 2,6-dihydroxy-3-methyl-4-pentylbenzaldehyde (**6**). Compound **6** was not isolated, but its NMR characterization was possible. ^1^H NMR (300 MHz, CDCl_3_): δ 12.51 (s, 1H), 10.06 (s, 1H), 6.52 (s, 1H), 6.23 (s, 1H), 2.91–2.78 (m, 2H), 2.12 (s, 3H), 1.64–1.49 (m, 2H), 1.38 (pd, *J* = 9.6, 8.1, 2.9 Hz, 4H), 0.91 (t, *J* = 6 Hz, 3H). ^13^C NMR (75 MHz, CDCl_3_): δ 193.66, 164.13, 162.41, 147.54, 115.53, 112.70, 100.71, 31.94, 31.58, 27.70, 22.47, 14.01, 10.41. mp: 120–123 °C. GCMS: *m*/*z* 222, *t*_R_: 10.5 min.

##### 1,3-Dihydroxy-4,6-dimethyl-5-pentylbenzene (**7**)

This compound was synthesized from 0.8 g (3.6 mmol) of **5** by the same method as described for **4**. Compound **7** was obtained as a white solid. Yield: 0.53 g (71%). mp: 136–138 °C. GCMS: *m*/*z* 208, *t*_R_: 10 min. ^1^H NMR (300 MHz, CDCl_3_, ppm): δ 6.19 (s, 1H), 4.74 (s, 2H), 2.59 (t, 2H), 2.13 (s, 6H), 1.74–1.19 (m, 6H), 0.93 (t, *J* = 6.3 Hz, 3H). ^13^C NMR (75 MHz, CDCl_3_): δ 151.94, 142.42, 114.23, 100.38, 32.42, 30.31, 29.28, 22.69, 14.25, 11.30. GCMS: *m*/*z* 208, *t*_R_: 10.05 min.

As (**5**) contained 10% of the secondary product, **6** in which the aldehyde is located between the two phenols, its reaction product was obtained as well and named herein 1,3-dihydroxy-2,4-dimethyl-6-pentylbenzene (**8**). It is possible to separate both compounds by chromatography. The secondary **8** is obtained at a yield of 73 mg (10%) and is an off-white solid. ^1^H NMR (300 MHz, CDCl_3_): δ 6.26 (s, 1H), 4.97 (s, 1H), 4.82 (s, 1H), 2.56–2.43 (m, 2H), 2.13 (s, 2H), 2.12 (s, 2H), 1.60–1.45 (m, 1H), 1.43–1.22 (m, 3H), 0.92 (t, *J* = 6 Hz, 3H). ^13^C NMR (75 MHz, CDCl_3_): δ 152.84, 151.86, 139.94, 113.48, 108.31, 107.31, 33.72, 31.93, 30.43, 22.72, 14.20, 11.16, 8.35. GCMS: *m*/*z* 208, *t*_R_: 9.82 min. mp: 100–101 °C.

##### 4′-Methylabnormalcannabidiol (**9**)

4′-Methylabnormalcannabidiol (**9**) was a secondary product in the synthesis of **1** from **4** and PMD (method 2). It was obtained from 1 g of **4** in a yield of 0.17 g (12%). The compound was code-named HUM-229; it was obtained as a pale-yellow oil. ^1^H NMR (300 MHz, CDCl_3_, ppm): δ 6.24 (1H, s), 6.07 (1H, s), 5.55 (1H, s), 5.39 (1H, s), 4.67 (1H, s), 4.49 (1H, s), 3.6 (1H, m), 2.52 (4H, m), 2.2 (2H, m), 2.12 (3H, s), 1.85–1.82 (2H, m), 1.84 (3H, s), 1.55 (3H, s), 1.45–1.35 (7H, m), 0.95–0.91 (3H, t, *J* = 6 Hz). ^13^C NMR (75 Hz, CDCl_3_, ppm): δ 153.54, 152.90, 147.70, 142.26, 139.61, 125.14, 120.12, 114.18, 111.40, 102.16, 44.90, 40.80, 32.37, 30.19, 30.12, 30.00, 27.99, 23.70, 22.55, 21.59, 14.16, 11.49. GCMS: *m*/*z* 328, *t*_R_: 12.3 min. [α]_D_ in EtOH: −87.8°.

##### 4′,6′-Dimethylcannabidiol (**10**)

4′,6′-Dimethylcannabidiol (**10**) was prepared from **7** and PMD in the same method described for **1** (method 2). It was obtained from 0.1 g (0.48 mmol) of **7** in a yield of 0.06 g (42%). The compound was code-named HUM-236; it was obtained as a pale-yellow oil. ^1^H NMR (300 MHz, CDCl_3_): δ 6.05 (s, 1H), 5.57 (s, 1H), 4.72 (s, 1H), 4.64 (s, 1H), 4.59 (s, 1H), 3.93–3.80 (m, 1H), 2.57 (t, *J* = 7.6 Hz, 2H), 2.41 (td, *J* = 10.6, 3.9 Hz, 1H), 2.30–2.17 (m, 1H), 2.11 (s, 4H), 1.87–1.82 (m, 1H), 1.80 (d, *J* = 2.3 Hz, 3H), 1.77–1.73 (m, 0H), 1.62 (s, 1H), 1.38 (s, 3H), 1.01–0.87 (m, 3H). ^13^C NMR (75 MHz, CDCl_3_): δ 150.68, 140.37, 139.52, 124.37, 116.05, 113.87, 113.53, 110.66, 45.74, 38.89, 32.44, 30.47, 30.26, 29.42, 28.70, 23.92, 22.71, 21.70, 14.26, 11.98, 11.40. GCMS: *m*/*z* 342, *t*_R_: 12.7 min. [α]_D_ in EtOH: −31.2°.

##### 4-Methylcannabigerol (**11**)

0.2 g (1.03 mmol) of **4** were dissolved in 3.3 mL of dry DCM with 0.017 g (0.103 mmol) of PTSA. This solution was then cooled to 0 °C. Separately, 0.17 mL (1.03 mmol) of geraniol were dissolved in 2.6 mL of dry DCM and then cooled to 0 °C as well. The cold geraniol solution was then added dropwise with high stir to the cold solution of **4**. The reaction was then stirred at RT for 45 min and quenched by the addition of a sat. NaHCO_3_ solution. The water phase was then separated from the organic phase, and the former was further extracted with DCM three times. The combined organic phase was washed with brine, dried over MgSO_4_ and evaporated. The crude product was then purified by silica gel column chromatography (TLC 20% EtOAc/hexanes). The **11** obtained from the column was further purified by recrystallization from pentane at −20 °C overnight. The solids were washed with cold pentane and dried on vacuum. The compound was code-named HUM-218; it was obtained as a white solid. Yield: 0.08 g (22%). ^1^H NMR (300 MHz, CDCl_3_, ppm): δ 6.25 (1H, s), 5.25 (1H, m), 5.06 (1H, m), 4.84 (1H, s), 3.43 (2H, d, *J* = 6.9 Hz), 2.50 (2H, t, *J* = 8.4 Hz), 2.1 (7H, m), 1.83 (3H, s), 1.69 (3H, s), 1.60 (3H, s), 1.52 (2H, m), 1.34 (4H, m), 0.90 (3H, t, *J* = 3.6 Hz); ^13^C NMR (75 MHz, CDCl_3_, ppm): δ 153.43, 151.66, 139.16, 132.16, 123.70, 121.81, 114.46, 110.44, 108.43, 39.71, 33.59, 31.86, 30.20, 26.29, 25.73, 22.68, 22.62, 17.73, 16.16, 14.10, 10.94. mp: 48–50 °C. GCMS: *m*/*z* 330, *t*_R_: 13.4 min.

##### 2-Methylcannabichromene (**13**) and 4-Methylcannabichromene (**14**)

0.5 g (2.58 mmol) of **2** and 0.5 mL of citral (3.1 mmol, mixture of cis and trans isomers) were dissolved in 30 mL of anhydrous toluene under a nitrogen atmosphere at RT. 93 mg (0.52 mmol) of ethylendiamine diacetate (EDDA) were added to the solution still at room temperature. A water condenser was then attached, and the reaction was refluxed and monitored by TLC (5% EtOAc in hexanes). After 6 h, the reaction was cooled to RT on an ice bath. The toluene was removed by vacuum evaporation to yield dark-orange oily crude. Both products were purified by silica gel column chromatography (TLC 5% EtOAc/hexanes). First, **13** was eluted from the column. It was code-named HUM-237 and was obtained as dark red oil. Yield: 0.18 g (21.4%). ^1^H NMR (500 MHz, CDCl_3_): δ 6.64 (d, *J* = 9.9 Hz, 1H), 6.30 (s, 1H), 5.52 (d, *J* = 9.9 Hz, 1H), 5.11 (t, *J* = 7.1 Hz, 1H), 4.79 (s, 1H), 2.54–2.48 (m, 2H), 2.13 (s, 2H), 2.09 (s, 4H), 1.74 (s, 2H), 1.68 (s, 3H), 1.59 (s, 3H), 1.54 (s, 3H), 1.39 (s, 4H), 1.36 (d, *J* = 7.2 Hz, 5H), 0.91 (s, 4H). ^13^C NMR (126 MHz, CDCl_3_): δ 151.46, 149.35, 142.71, 131.65, 127.79, 124.37, 117.18, 113.01, 109.64, 107.22, 77.85, 41.03, 34.10, 32.06, 31.92, 30.18, 29.84, 26.18, 25.79, 22.84, 22.70, 17.74, 14.16, 10.84, 1.15. GCMS: *m*/*z* 328, *t*_R_: 12.45 min.

Second, **14** was eluted from the column. It was code-named HUM-238 and was obtained as dark brown oil. Yield: 0.17 g (20%). ^1^H NMR (500 MHz, CDCl_3_): δ 6.65 (d, *J* = 5 Hz, 1H), 6.14 (s, 1H), 5.52 (d, *J* = 10 Hz, 1H), 5.13 (t, *J* = 5 Hz, 2H), 4.98 (s, 1H), 2.48 (t, *J* = 10 Hz, 2H), 2.2–2.1 (m, 3H), 2.08 (s, 3H), 1.75–1.71 (m, 4H), 1.68 (s, 3H), 1.60 (s, 3H), 1.52 (t, *J* = 5 Hz, 3H), 1.40 (s, 3H), 1.37–1.34 (m, 4H), 0.92 (s, 3H). ^13^C NMR (126 MHz, CDCl_3_): δ 151.68, 148.85, 142.75, 131.64, 127.24, 124.46, 117.31, 115.95, 107.97, 107.11, 77.97, 77.16, 41.02, 33.85, 31.98, 30.15, 26.10, 25.79, 22.86, 22.73, 17.66, 14.18, 10.55. GCMS: *m*/*z* 328, *t*_R_: 12.44 min.

##### 4′-Methyldimethoxycannabidiol (**15**)

0.1 g (0.3 mmol) of **1** were dissolved in 0.5 mL of dry DMF under a nitrogen atmosphere. The solution was added to 5 mL suspension of 0.17 g (1.22 mmol) potassium carbonate in dry DMF. The reaction was stirred for 5 min at room temperature. Then, 230 μL (3.65 mmol) of iodomethane were added to the reaction. The reaction was heated to 40 °C and stirred overnight. The reaction was quenched by adding 10% w/v HCl to pH 1. The aqueous phase was extracted with EtOAc, dried over MgSO_4_ and evaporated. The crude product was purified by thick layer chromatography plate (1.25% ether/hexanes). The compound was code-named HUM-219; it was obtained as a colorless oil. Yield: 0.03 g (28%). ^1^H NMR (300 MHz, CDCl_3_, ppm): δ 6.45 (1H, s), 5.26 (1H, s), 4.47 (2H, s), 3.9 (1H, s), 3.72 (3H, s), 3.62 (3H, s), 2.96 (1H, td, *J* = 15.6, 3.6 Hz), 2.52 (2H, t, *J* = 8.1 Hz), 2.17 (1H, m), 2.13 (3H, s), 2.01 (1H, m), 1.76 (2H, m), 1.68 (3H, s), 1.54 (6H, m), 1.36 (4H, t, *J* = 3.3 Hz), 0.91 (3H, t, *J* = 6.3 Hz); ^13^C NMR (75 MHz, CDCl_3_, ppm): δ 149.48, 123.89, 109.79, 80.70, 60.91, 56.02, 45.02, 33.99, 31.99, 30.75, 29.94, 29.67, 23.52, 22.59, 19.52, 14.07, 11.72. GCMS: *m*/*z* 356, *t*_R_: 11.46 min. [α]_D_ in EtOH: −118.8°.

##### 4-Methyldimethoxycannabigerol (**16**)

0.132 g (0.4 mmol) of **11** were dissolved in a suspension of 0.22 g (1.6 mmol) of potassium carbonate in 1 mL of dry acetone under a nitrogen atmosphere at room temperature. To this suspension was added 133 μL (1.4 mmol) of dimethyl sulfate. A water condenser was attached, and the reaction was refluxed overnight. The suspension was filtered, and the acetone was evaporated. The crude was then partition between EtOAc and sat. NaHCO_3_ solution, and the water phase was extracted three times with EtOAc. The combined organic phase was then washed with 10% w/v HCl, sat. NaHCO_3_ and brine, dried over magnesium sulfate, filtered, and evaporated. **16** that was obtained after this workup was sufficiently pure and did not require further purification. The compound was code-named HUM-235; it was obtained as a clear colorless oil. Yield: 0.13 g (88%). ^1^H NMR (300 MHz, CDCl_3_): δ 6.54 (s, 1H), 5.25 (t, *J* = 6.4 Hz, 1H), 5.11 (t, *J* = 6.7 Hz, 1H), 3.84 (s, 3H), 3.72 (s, 3H), 3.39 (d, *J* = 6.8 Hz, 2H), 2.68–2.52 (m, 2H), 2.22 (s, 3H), 2.09 (t, *J* = 7.3 Hz, 2H), 2.06–1.97 (m, 2H), 1.82 (d, *J* = 1.4 Hz, 3H), 1.68 (d, *J* = 1.5 Hz, 3H), 1.67–1.54 (m, 3H), 1.50–1.36 (m, 2H), 0.96 (t, *J* = 4.7 Hz, 3H). ^13^C NMR (75 MHz, CDCl_3_): δ 157.24, 156.09, 140.42, 134.30, 131.13, 124.51, 123.75, 121.01, 120.72, 107.56, 60.69, 55.66, 39.82, 34.10, 32.04, 30.30, 26.73, 25.71, 23.12, 22.67, 17.68, 16.11, 14.14, 11.47. GCMS: *m*/*z* 358, *t*_R_: 12.25 min.

#### Animals

All experimental procedures were approved by the Ethics Review Process Committee and the UK Home Office in accordance with the 1986 Animals (Scientific Procedures) Act (permission no. 30/3441) or by the Institutional Animal Care Ethics Committee (permission no. MD-20-16042-5).

For zymosan-induced inflammation, female Sabra mice, 7–8 weeks old, were maintained in the specific-pathogen-free unit of the Hadassah Medical School, Hebrew University, Jerusalem, Israel. The animals were maintained at a constant temperature (20–21 °C) and a 12 h light/12 h dark cycle and were provided a standard pellet diet with water ad libitum. The mice were acclimatized in the animal facility for at least 2 weeks before the experiments. The data presented in figures are representative of 2 separate experiments. For the peritonitis experiments, C57Bl/6 were purchased from Envigo (Bicester, UK) and acclimatized for at least 1 week prior to initiation of experiments.

##### Induction and Treatment of Paw Inflammation—Zymosan-Induced Inflammation

Inflammation was induced by injection of 40 μL of a suspension of 1.5% w/v zymosan A (Sigma-Aldrich Israel Ltd., Rehovot, Israel) in saline into the subplantar surface of the right hind paw of the mice. This was followed immediately by an intraperitoneal injection of the test compound. For injection, the compounds were dissolved in a vehicle containing ethanol/Cremophor/saline at a ratio of 1:1:18. Paw swelling and pain perception were assessed after 2, 6, and 24 h. Blood was collected after 24 h for analysis of TNFα serum levels.

##### Evaluation of Edema

Calibrated calipers were used to measure paw swelling (thickness) 2, 6, and 24 h after injection of zymosan.

##### Pain Assay

Pain at 2, 6, and 24 h after zymosan injection was assessed by the von Frey nociceptive filament assay, where 1.4–60 g filaments, corresponding to 4.17–5.88 log of force, were used to test the pain response to pressure of the swollen paw. The untreated hind paw served as a control. The measurements were performed in a quiet room, and the animals were handled for 10 s before the test. A trained investigator then applied the filament, poking the middle of the hind paw to provoke a flexion reflex, followed by a clear finch response after paw withdrawal. Filaments of increasing size were each applied for about 3–4 s. The mechanical threshold force in grams was defined as the lowest force required to obtain a paw retraction response.

##### Measurement of TNFα

Blood was collected 24 h after zymosan injection, and the sera were assayed for TNFα using a mouse TNFα ELISA kit (R&D Systems, Minneapolis, MN, USA), according to the manufacturer’s instructions.

##### LPS-Induced Peritonitis

With five per treatment group, 8 week old C57BL/6 mice were injected intraperitoneally with test compounds **2**, **11,** or **15**, dexamethasone or vehicle only, 60 min prior to an intraperitoneal injection of 2.5 mg/kg LPS. After a further 90 min, blood was collected humanely from euthanized mice via cardiac puncture. Plasma was extracted and frozen at −80 °C for subsequent cytokine analysis by ELISA. Cytokines TNFα, IL-β, and IL-6 were quantified using the MSD platform, according to the manufacturer’s instructions.

##### Human Blood Monocyte-Derived Macrophages

Human blood monocyte-derived macrophages were pre-treated with vehicle, CBD, or **2** for 3 h, then stimulated with 100 ng/mL LPS (Sigma, L2880) for 3 h, followed by 4 mM ATP (Sigma, A2383) for 30 min. Supernatants were then collected for measurement of IL-1β by ELISA (Thermo Scientific, 88-7013), and macrophage cell lysates were collected for qRT-PCR. The housekeeping gene *HPRT1* (Thermo Scientific, Hs99999909_m1) was used as the baseline to quantify the expression of *IL1B* (Thermo Scientific, Hs01555410_m1).

## Supporting Information Available

HSQC, HMBC, 2D-COSY, and 2D-NOESY spectra of **13**; comparison of 1D-NOESY and ^1^H NMR spectra of **13**; HSQC, HMBC, 2D-COSY, and 2D-NOESY spectra of **14**; comparison of ^1^H NMR spectra of **14** in CDCl_3_ in the presence and absence of D_2_O; 1D-NOESY spectrum of **14**; GCMS chromatogram and purity report for **1**, **2**, **9**, **10**, **11**, **13**, **14**, **15**, and **16** (PDF)
Code names and SMILES for compounds (CSV)

## Dedication

This article is dedicated to the memory of Professor Raphael Mechoulam (1930–2023), who directed this project until his passing.

## Abbreviations

CBC: cannabichromene
CBD: cannabidiol
CBDA: cannabidiolic acid
CBG: cannabigerol
DCM: methylene chloride
DMF: dimethylformamide
EDDA: ethylenediamine diacetate
IL-1β: interleukin 1-β
IL-6: interleukin
LiAlH_4_: lithium aluminum hydride
LPS: lipopolysaccharides
MMC: methyl magnesium carbonate
NSAIDs: non-steroidal anti-inflammatory drugs
PMD: (1*S*,4*R*)-*p*-mentha-2,8-dien-1-ol
PTSA: *p*-toluenesulfonic acid
RA: rheumatoid arthritis
Red-Al: lithium bis(2-methoxyethoxy)aluminum hydride
THF: tetrahydrofuran
TNFα: tumor necrosis factor-α
THC: Δ^9^-tetrahydrocannabinol
