# Supplementary material for: Novel Synthesis of C-Methylated Phytocannabinoids Bearing Anti-inflammatory Properties
Source: J Med Chem. 2023 Apr 14;66(8):5536–49. doi: 10.1021/acs.jmedchem.2c01988 (PMC10150364; doi:10.1021/acs.jmedchem.2c01988)
Supplement: Supplementary file 1 — jm2c01988_si_001.pdf [file jm2c01988_si_001.pdf]

Supporting Information

Novel synthesis of C-methylated phytocannabinoids bearing anti-inflammatory properties

Yarden Lavi[1]‡†, Natalya M. Kogan[2,1]‡*, Louise M. Topping[3], Caojie Liu[3], Fiona E. McCann[4], Richard. O. Williams[3], Aviva Breuer[1], Zhanna Yekhtin[5], Aviva Friedman-Ezra[1], Ruth Gallily[5], Marc Feldmann[3,4] and Raphael Mechoulam[1]

[1] Medicinal chemistry, Institute of drug research, The Hebrew University of Jerusalem, 91120, Jerusalem, Israel

[2] Institute of Personalized and Translational Medicine, Molecular Biology, Ariel University, 4070000, Ariel, Israel

[3] Kennedy Institute of Rheumatology, University of Oxford, Oxford OX3 7FY, UK

[4] 180 Life Sciences, Menlo Park, CA 94025, USA

[5] Lautenberg center of immunology and cancer research, The Hebrew University of Jerusalem, 91120, Jerusalem, Israel

‡ - equally contributed

* - Correspondence: natalyak@ariel.ac.il

Table of contents

Table S1 Code names and SMILES for compounds **3**

2D NMR spectra of compounds 13 and 14 **4-9**

Figure S1 HSQC experiment of (13)

Figure S2 HMBC experiment of (13)

Figure S3 2D-COSY of (13)

Figure S4 2D-NOSEY of (13)

Figure S5 1D-NOSEY of (13) in Red and ^{1}H NMR in Blue. The focus is on the phenolic proton. The conclusion is illustrated.

Figure S6 HSQC experiment of (14).

Figure S7 HMBC experiment of (14)

Figure S8 2D-COSY of (14)

Figure S9 2D-NOESY of (14)

Figure S10 ^{1}H NMR of (14) with focus between 4.0 and 7.5 ppm. Comparison between $CDCl_3$ and $CDCl_3$+D_2O

Figure S11 1D-NOSEY of (14) coupled to 1H NMR of the compound. The focus is on the aromatic proton.

GCMS and purity reports of phytocannabinoid derivatives **10-18**

Figure S12 GCMS and purity reports of **(1)**

Figure S13 GCMS and purity reports of **(2)**

Figure S14 GCMS and purity reports of **(9)**

Figure S15 GCMS and purity reports of **(10)**

Figure S16 GCMS and purity reports of **(11)**

Figure S17 GCMS and purity reports of **(13)**

Figure S18 GCMS and purity reports of **(14)**

Figure S19 GCMS and purity reports of **(15)**

Figure S20 GCMS and purity reports of **(16)**

Compound	Code name	SMILES
**1**	**HUM-216**	OC1=C(C)C(CCCCC)=CC(O)=C1[C@@H]2C=C(C)CC[C@H]2C(C)=C
**2**	**HUM-217**	OC1=C(C)C(CCCCC)=CC(O)=C1[C@@H]2C=C(C)CC[C@H]2C(C)C
**3**		OC(C=C1CCCCC)=CC(O)=C1[R]
**4**		CC1=C(C=C(C=C1CCCCC)O)O
**5**		OC(C(C)=C1CCCCC)=CC(O)=C1C=O
**6**		OC1=C(C=O)C(O)=CC(CCCCC)=C1C
**7**		CC1=C(C=C(C(C)=C1CCCCC)O)O
**8**		OC1=C(C)C(O)=CC(CCCCC)=C1C
**9**	**HUM-229**	CC1=C[C@@H](C2=C(O)C=C(O)C(C)=C2CCCCC)[C@H](C(C)=C)CC1
**10**	**HUM-236**	OC1=C(C)C(CCCCC)=C(C)C(O)=C1[C@@H]2C=C(C)CC[C@H]2C(C)=C
**11**	**HUM-218**	OC1=C(C)C(CCCCC)=CC(O)=C1C/C=C(C)/CC/C=C(C)\C
**12**		CC(C(CCCCC)=C(C/C=C(C)/CC/C=C(C)\C)C(O)=C1)=C1O
**13**	**HUM-237**	C/C(C)=C/CCC1(C)OC2=CC(CCCCC)=C(C)C(O)=C2C=C1
**14**	**HUM-238**	C/C(C)=C/CCC1(C)OC2=C(C)C(CCCCC)=CC(O)=C2C=C1
**15**	**HUM-219**	CC(CC[C@H]1C(C)=C)=C[C@H]1C2=C(OC)C=C(CCCCC)C(C)=C2OC
**16**	**HUM-235**	C/C(CC/C=C(C)/C)=C\CC1=C(OC)C=C(CCCCC)C(C)=C1OC

Table S1. Code names and SMILES for compounds.

2D NMR spectra of compounds 13 and 14

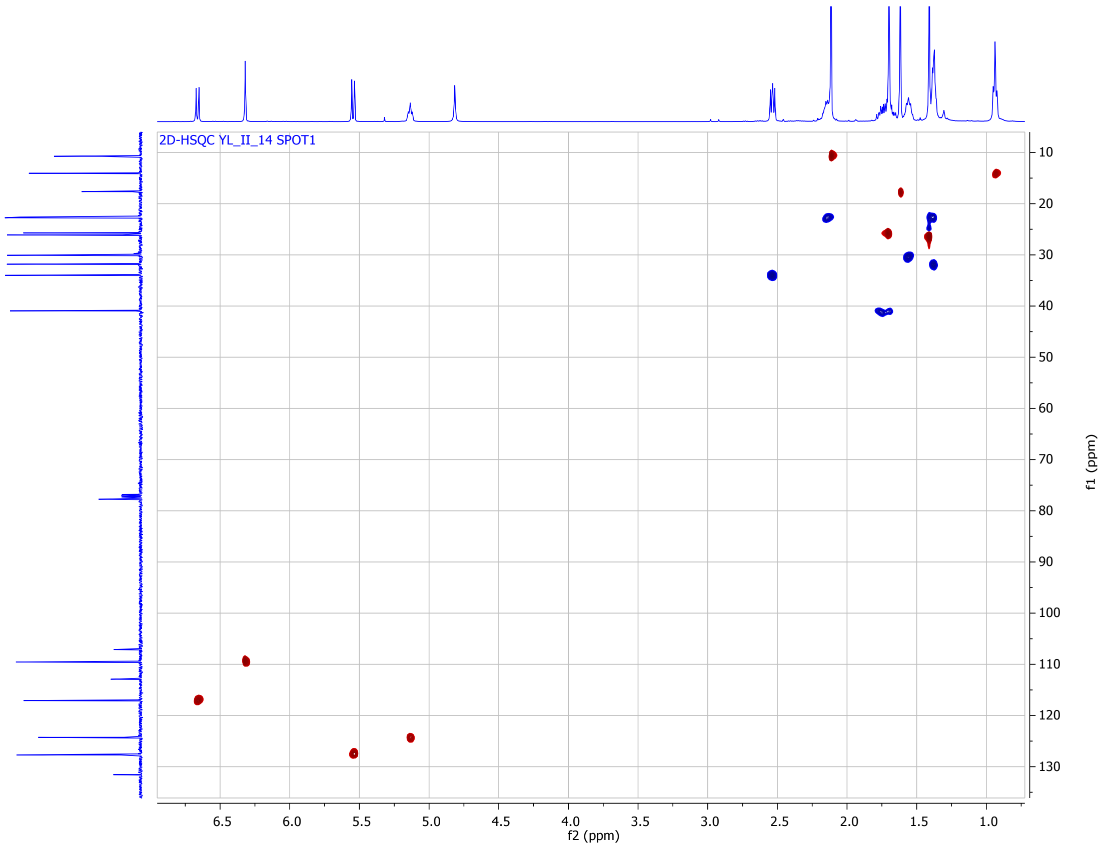

Figure S1. HSQC experiment of (13). Odd protons in red and even numbers in blue.

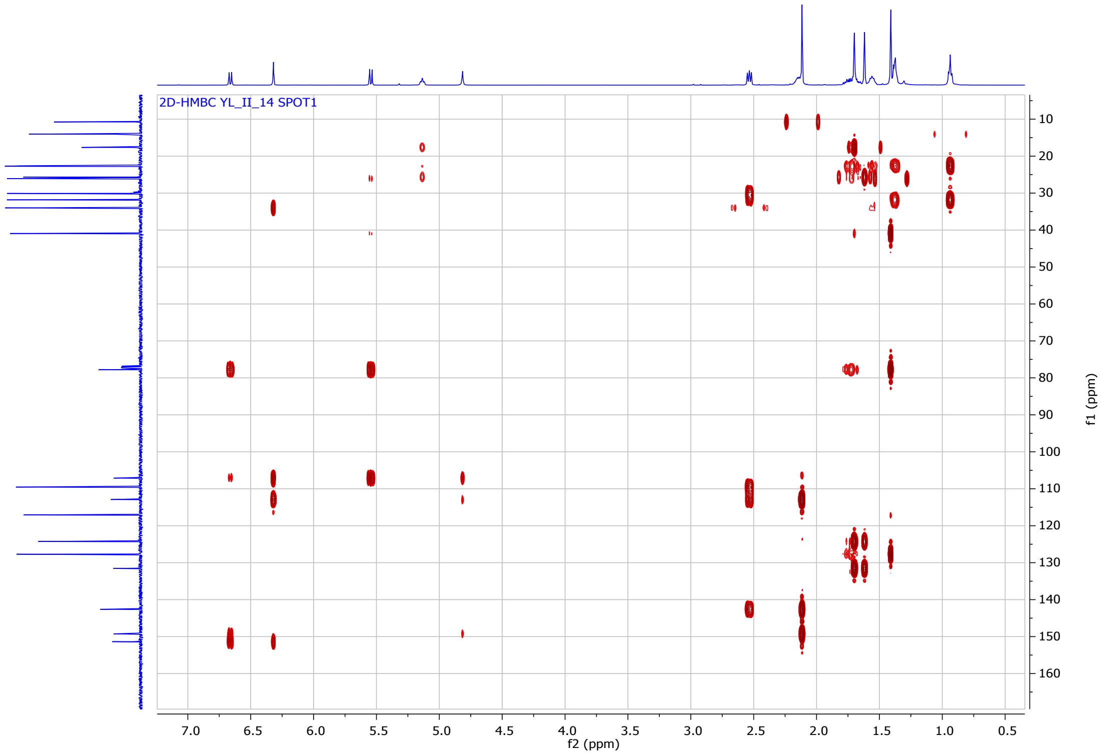

Figure S2. HMBC experiment of (13)

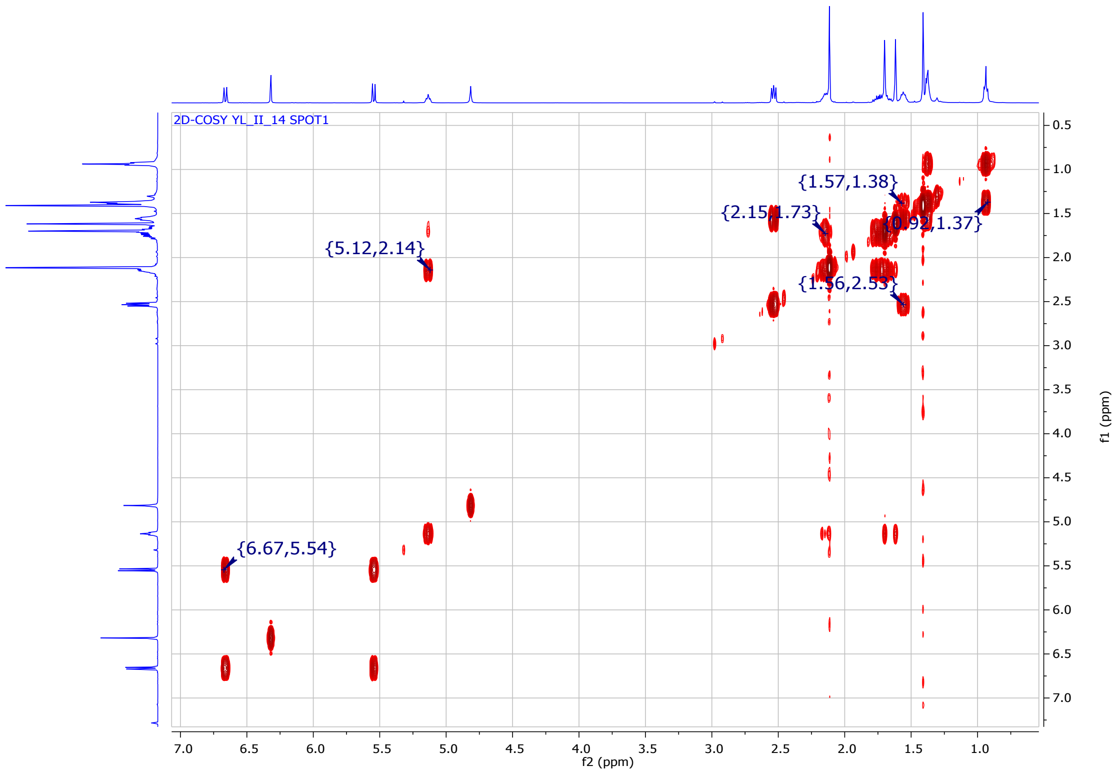

Figure S3. 2D-COSY of (13)

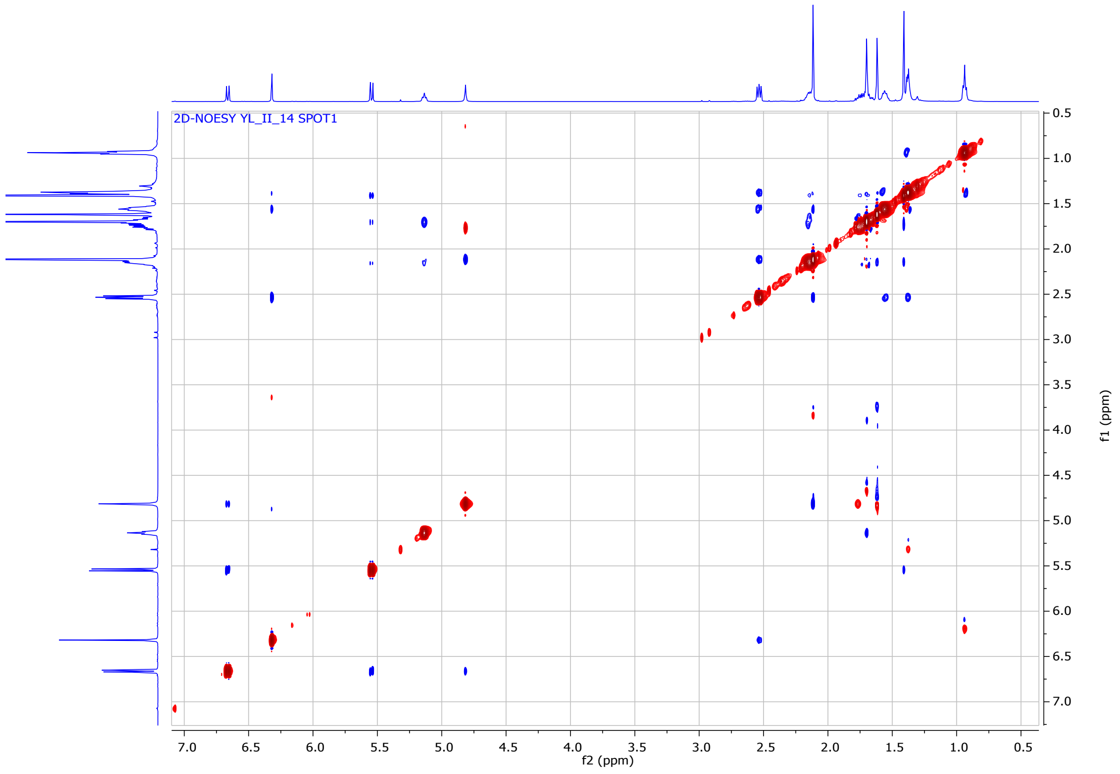

Figure S4. 2D-NOSEY of (13)

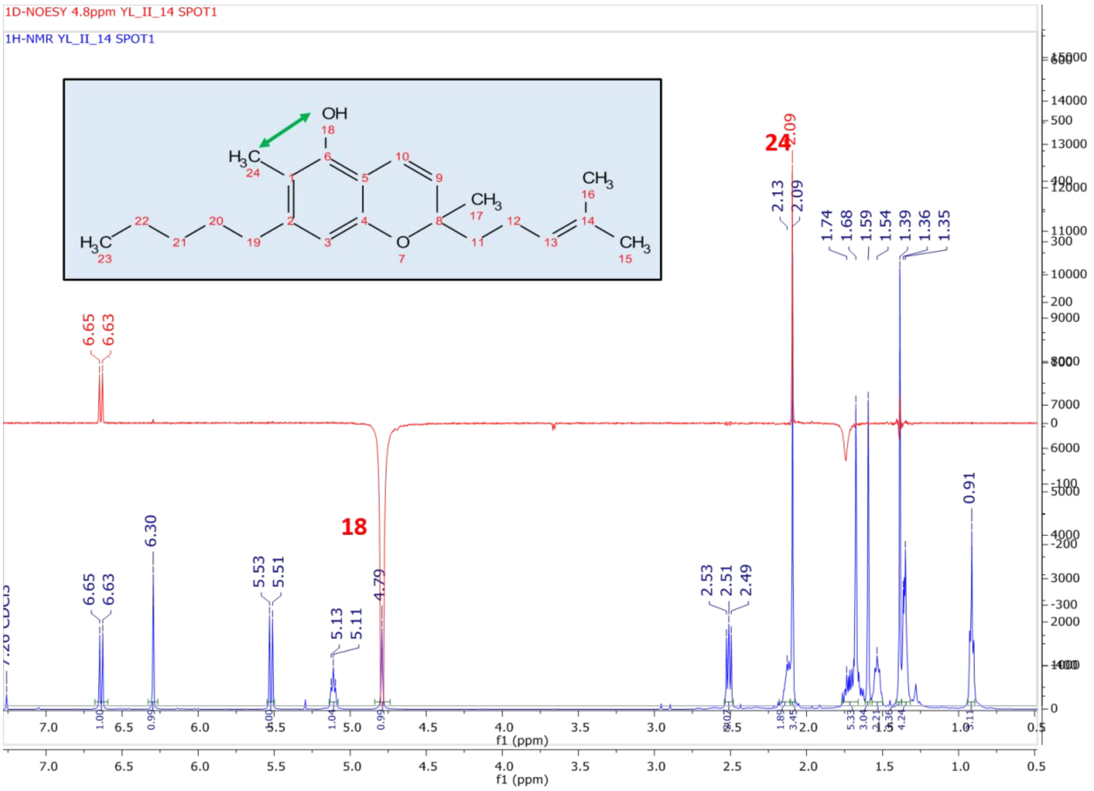

Figure S5. 1D-NOSEY of (13) in Red and ^{1}H NMR in Blue. The focus is on the phenolic proton (pick is turning downwards). The highlighted protons after the pulse are turning upwards. The conclusion is illustrated on the structure as shown in the green arrow.

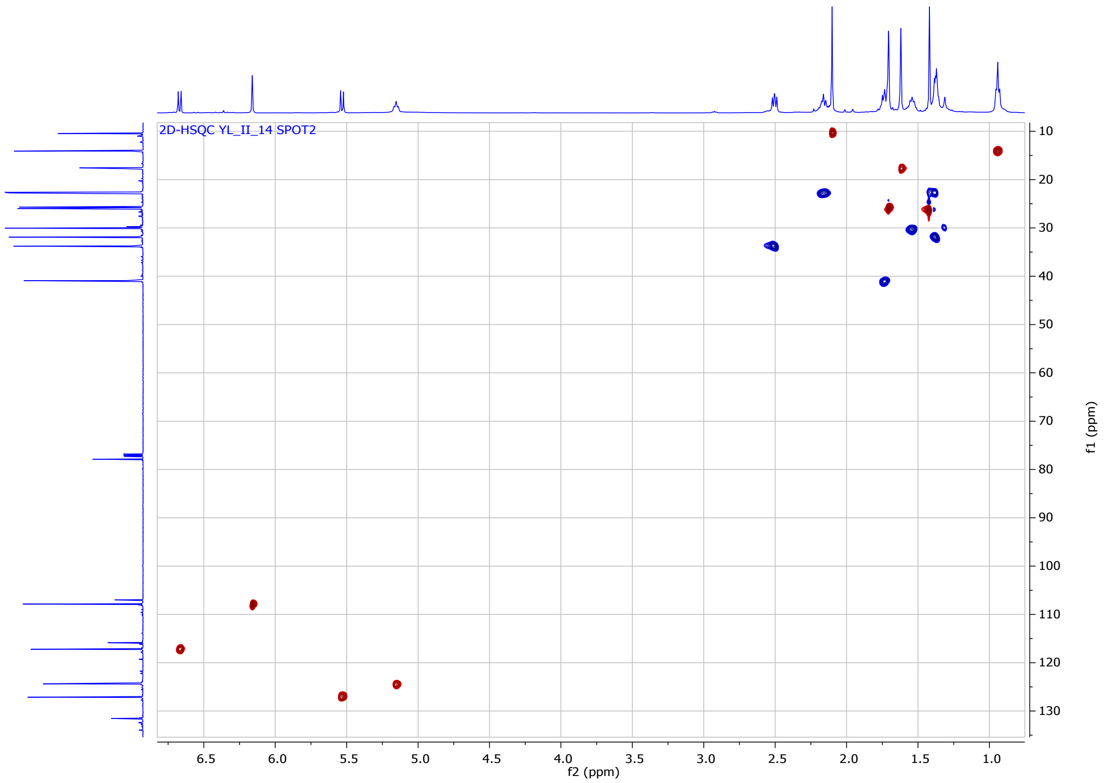

Figure S6. HSQC experiment for (14). Odd numbered nuclei in Red and even number nuclei in Blue.

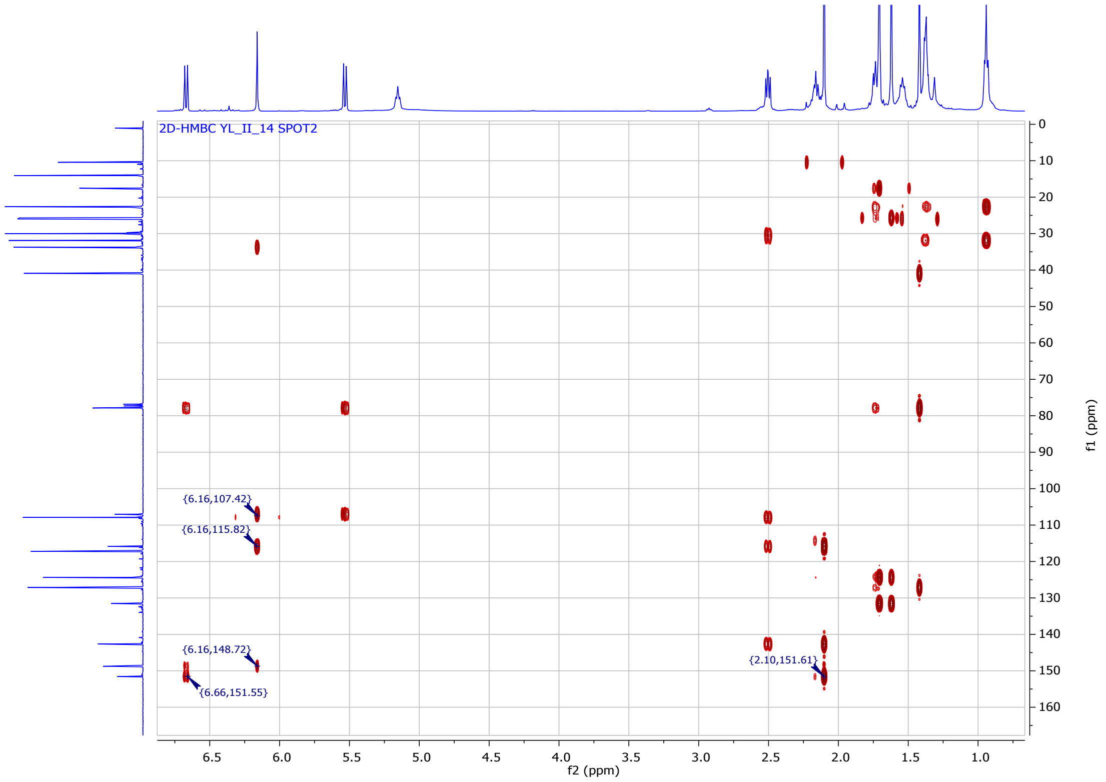

Figure S7. HMBC experiment of (14)

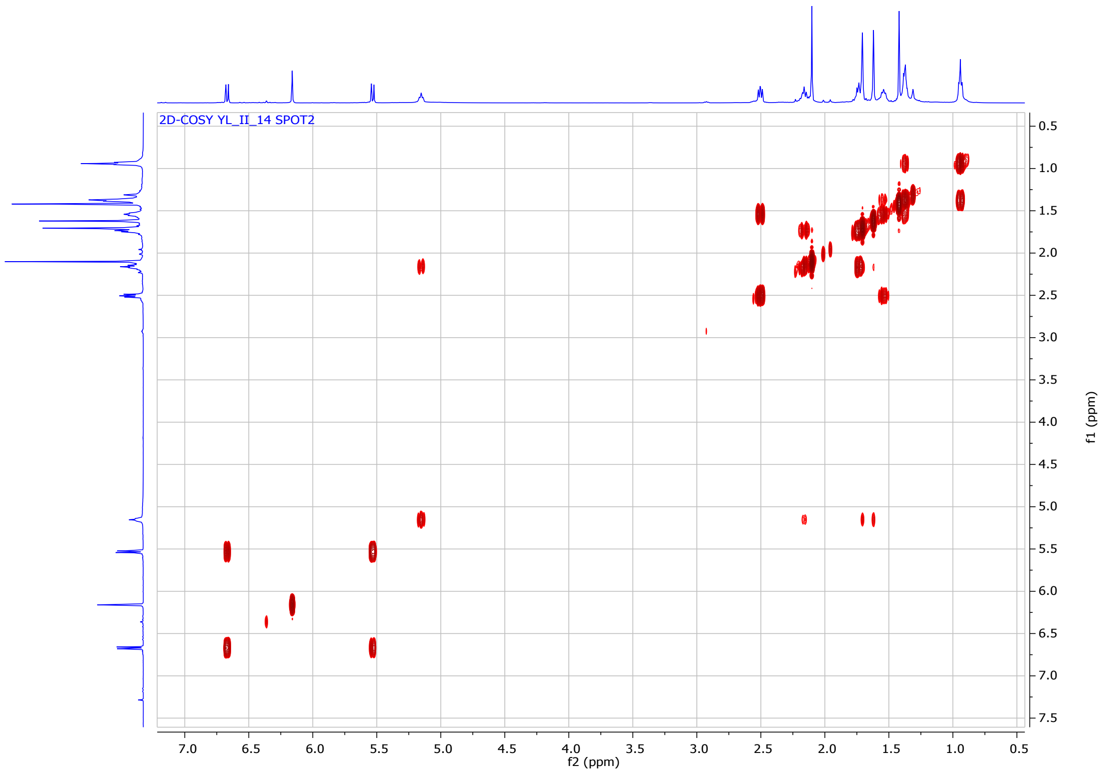

Figure S8. 2D-COSY of (14)

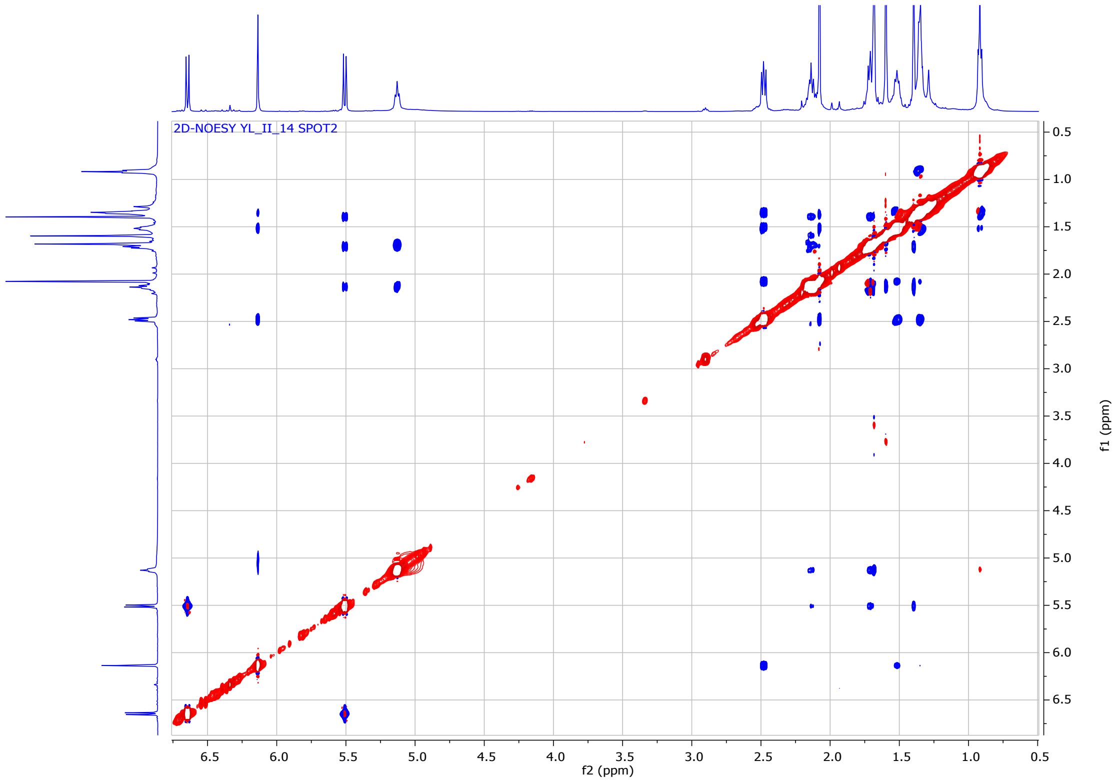

Figure S9. 2D-NOESY of (14)

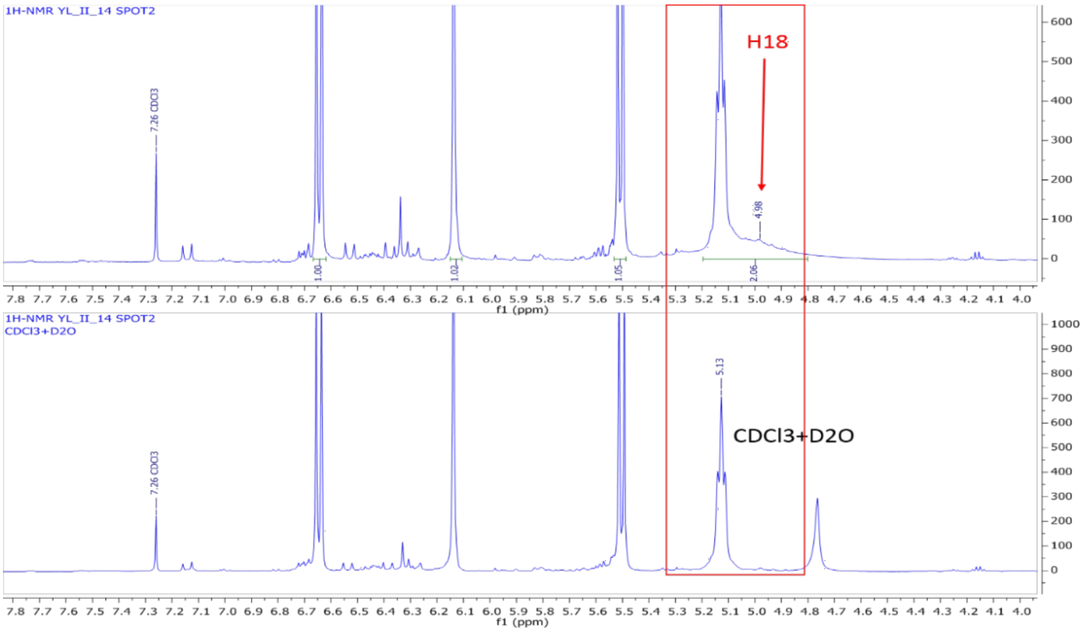

Figure S10. ^{1}H NMR of (14) with focus between 4.0 and 7.5 ppm. The upper spectrum is with $CDCl_3$ as solvent and the lower spectrum is with an addition of a small quantity of D_2O. Highlighted in the red rectangle is the phenolic proton.

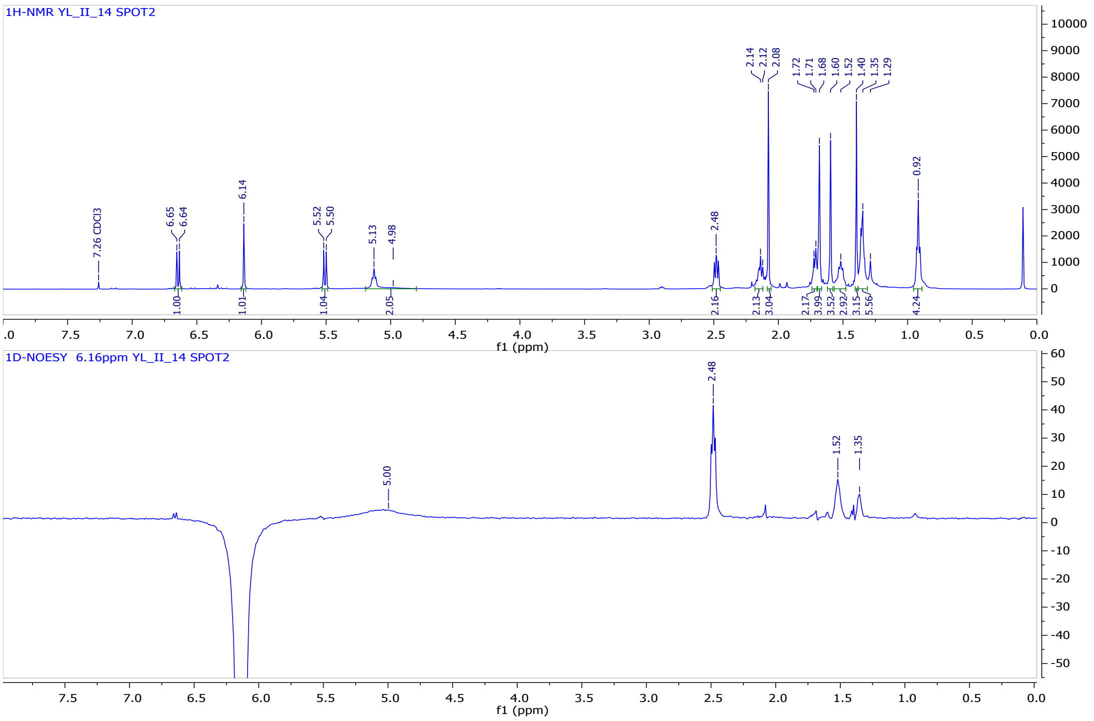

Figure S11. 1D-NOSEY of (14) coupled to ^{1}H NMR of the compound. The focus is on the aromatic proton (turning downwards)

GCMS and purity reports of phytocannabinoid derivatives

Compound (1)

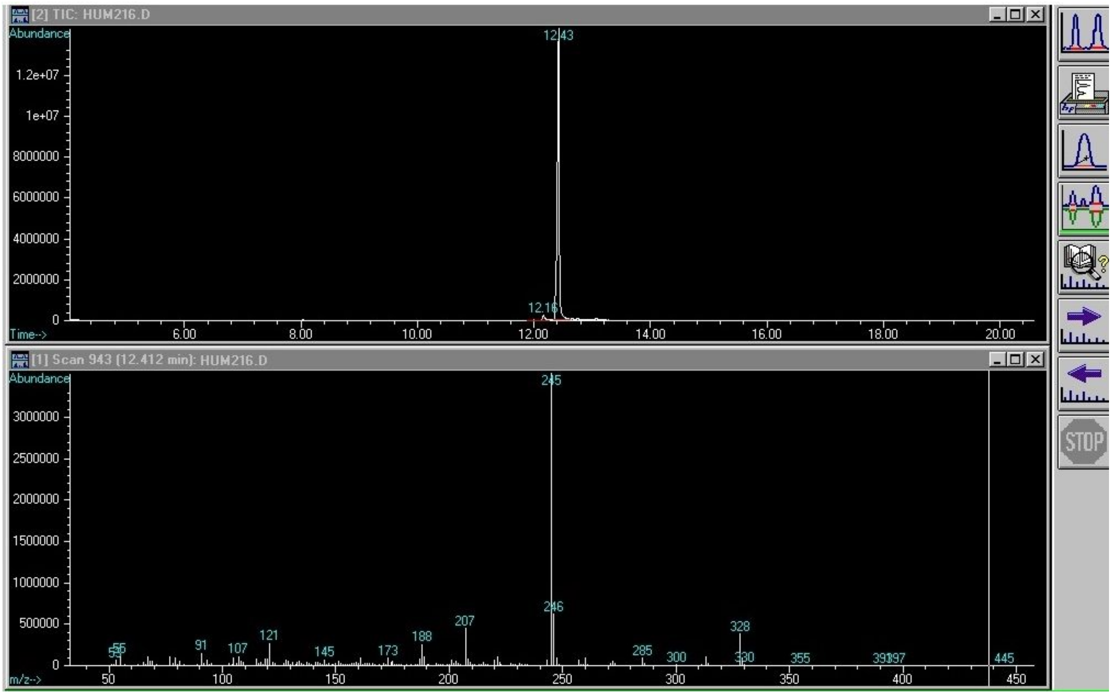

Figure S12. GCMS chromatogram and purity report for Compound 1

Area Percent Report -- Sorted by Signal

Retention Time	Area	Area %	Ratio %	Type	Width
Total Ion Chromatogram					
12.164	6385283	1.685	1.714	BV	0.041
12.428	372497825	98.315	100.000	PV	0.043

Figure S12. GCMS chromatogram and purity report for Compound 1

Compound (2)

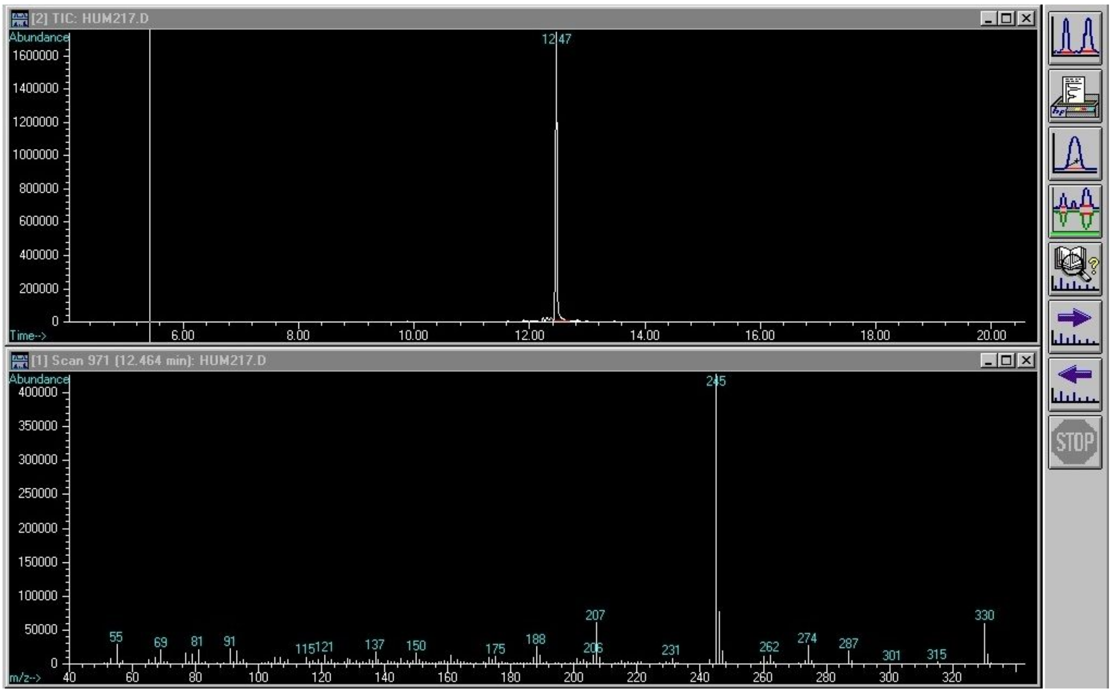

Area Percent Report -- Sorted by Signal

Retention Time	Area	Area %	Ratio %	Type	Width
Total Ion Chromatogram					
12.473	29927363	100.000	100.000	VB	0.049

Figure S13. GCMS chromatogram and purity report for Compound 2

Compound (9)

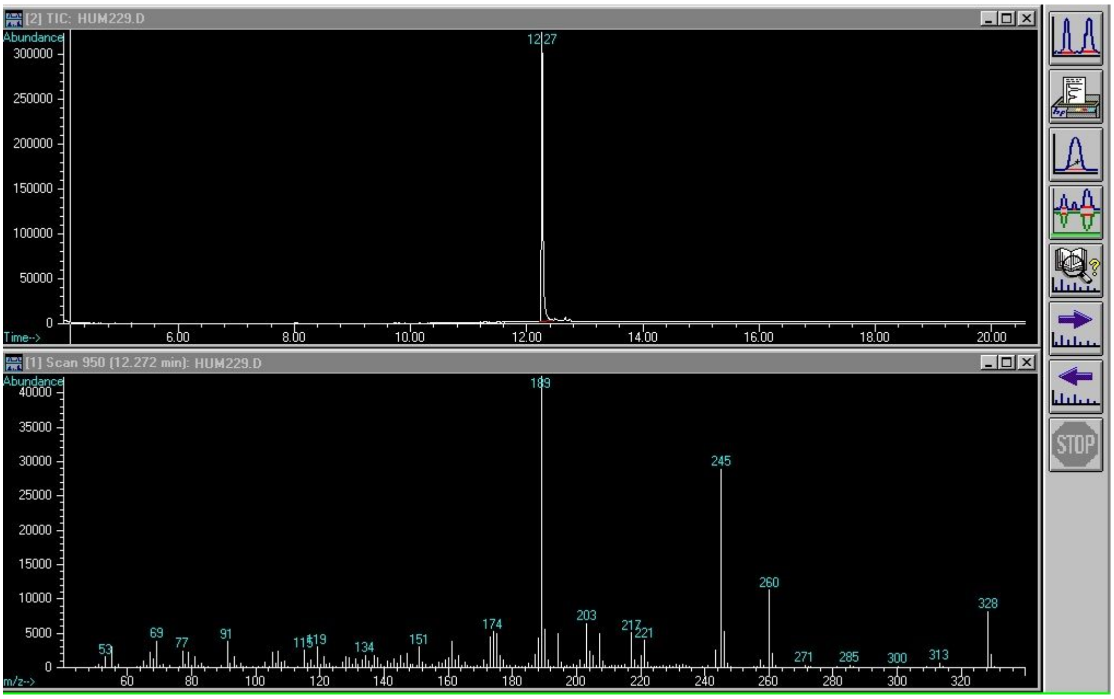

Area Percent Report -- Sorted by Signal

Retention Time	Area	Area %	Ratio %	Type	Width
Total Ion Chromatogram					
12.270	5766795	100.000	100.000	BV	0.029

Figure S14. GCMS chromatogram and purity report for Compound 9

Compound (10)

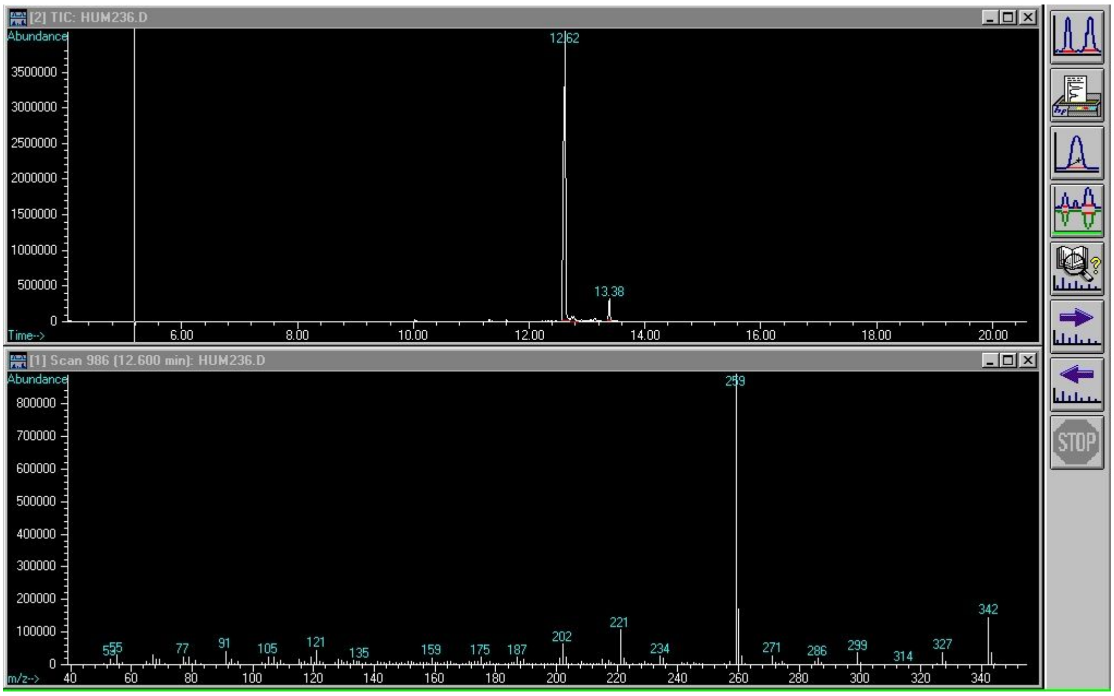

Area Percent Report -- Sorted by Signal

Retention Time	Area	Area %	Ratio %	Type	Width
Total Ion Chromatogram					
12.618	105032454	95.233	100.000	M	0.043
13.382	5257217	4.767	5.005	M	0.029

Figure S15. GCMS chromatogram and purity report for Compound 10

Compound (11)

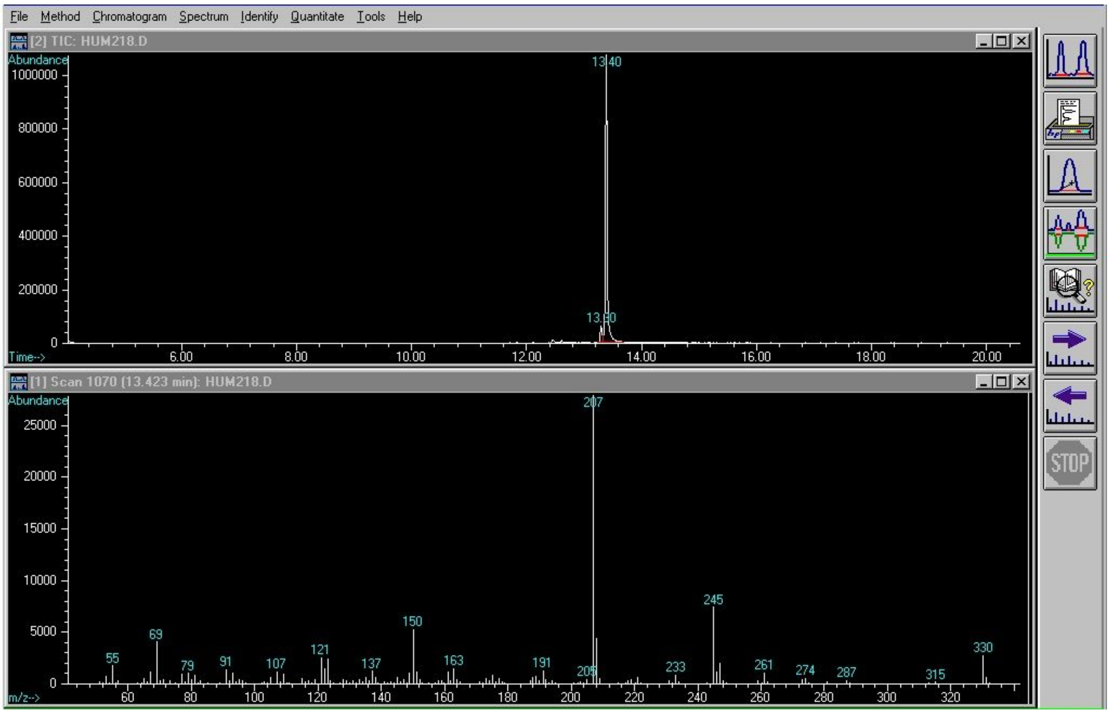

Area Percent Report -- Sorted by Signal

Retention Time	Area	Area %	Ratio %	Type	Width
Total Ion Chromatogram					
13.298	704229	3.022	3.116	M	0.027
13.393	22597523	96.978	100.000	M	0.035

Figure S16. GCMS chromatogram and purity report for Compound 11

Compound (13)

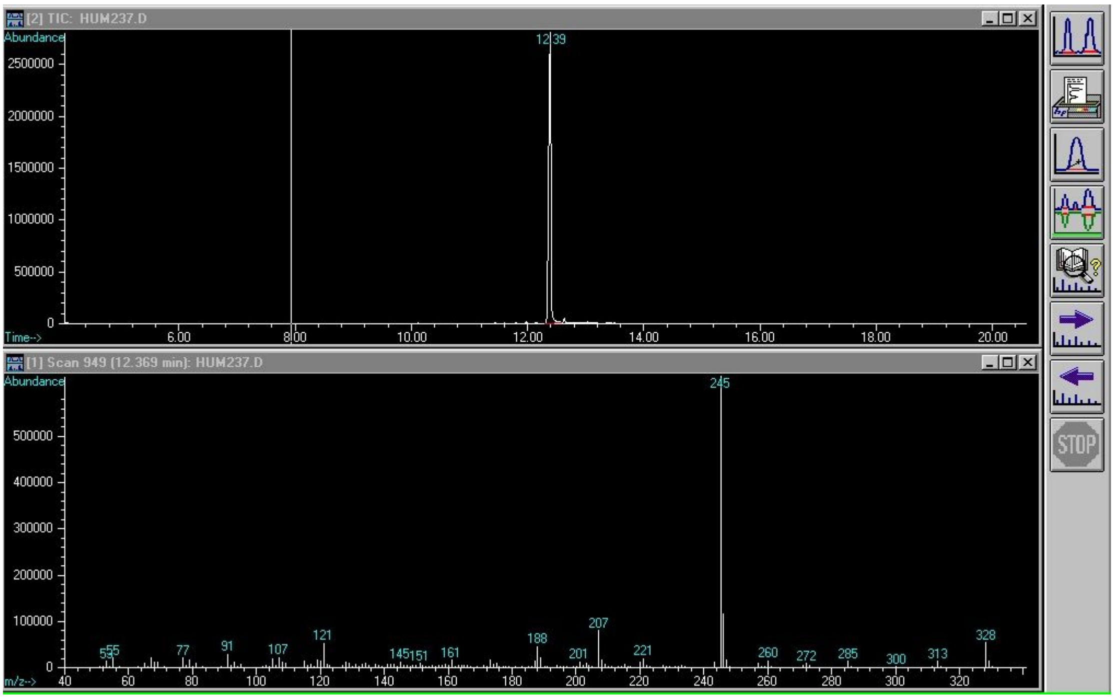

Area Percent Report -- Sorted by Signal

Retention Time	Area	Area %	Ratio %	Type	Width
Total Ion Chromatogram					
12.394	74981490	100.000	100.000	BV	0.045

Figure S17. GCMS chromatogram and purity report for Compound 13

Compound (14)

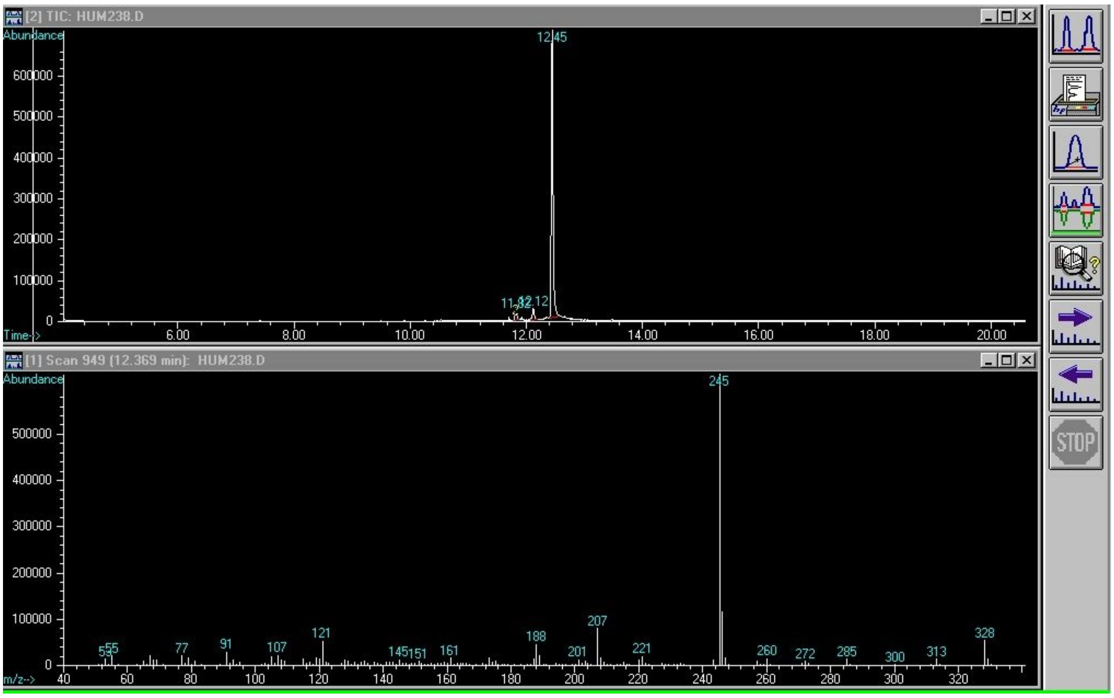

Area Percent Report -- Sorted by Signal

Retention Time	Area	Area %	Ratio %	Type	Width
Total Ion Chromatogram					
11.820	154692	1.061	1.111	BV	0.036
12.123	543096	3.795	3.973	BB	0.026
12.448	13881993	95.521	100.000	BV	0.031

Figure S18. GCMS chromatogram and purity report for Compound 14

Compound (15)

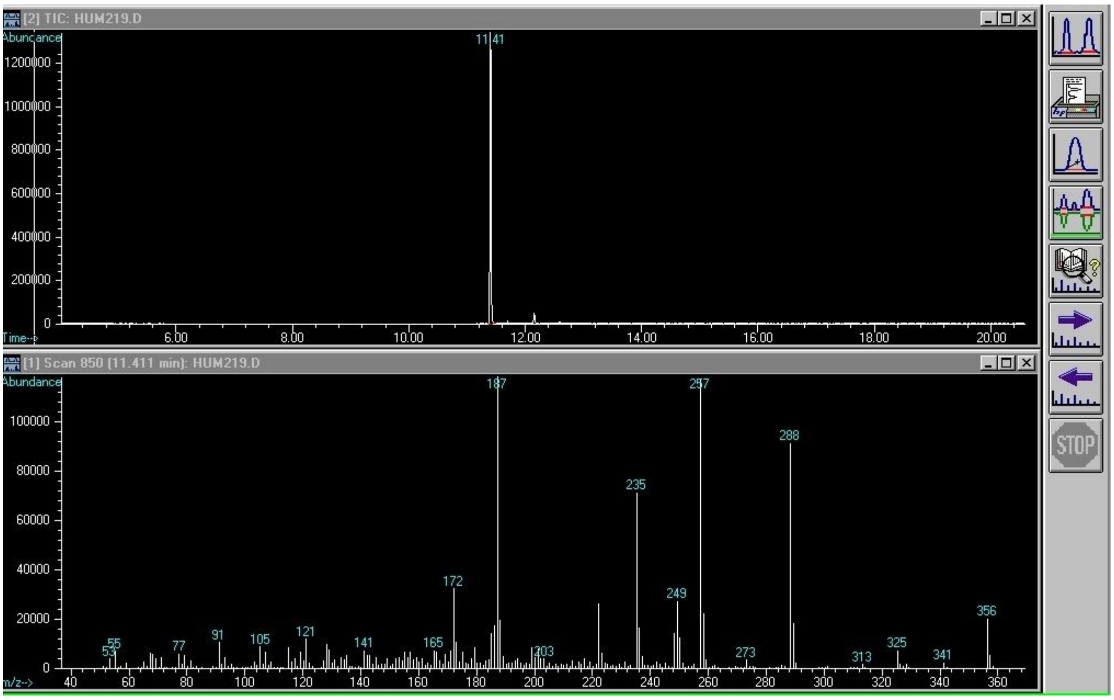

Area Percent Report -- Sorted by Signal

Retention Time	Area	Area %	Ratio %	Type	Width
Total Ion Chromatogram					
11.409	17137770	100.000	100.000	BB	0.022

Figure S19. GCMS chromatogram and purity report for Compound 15

Compound (16)

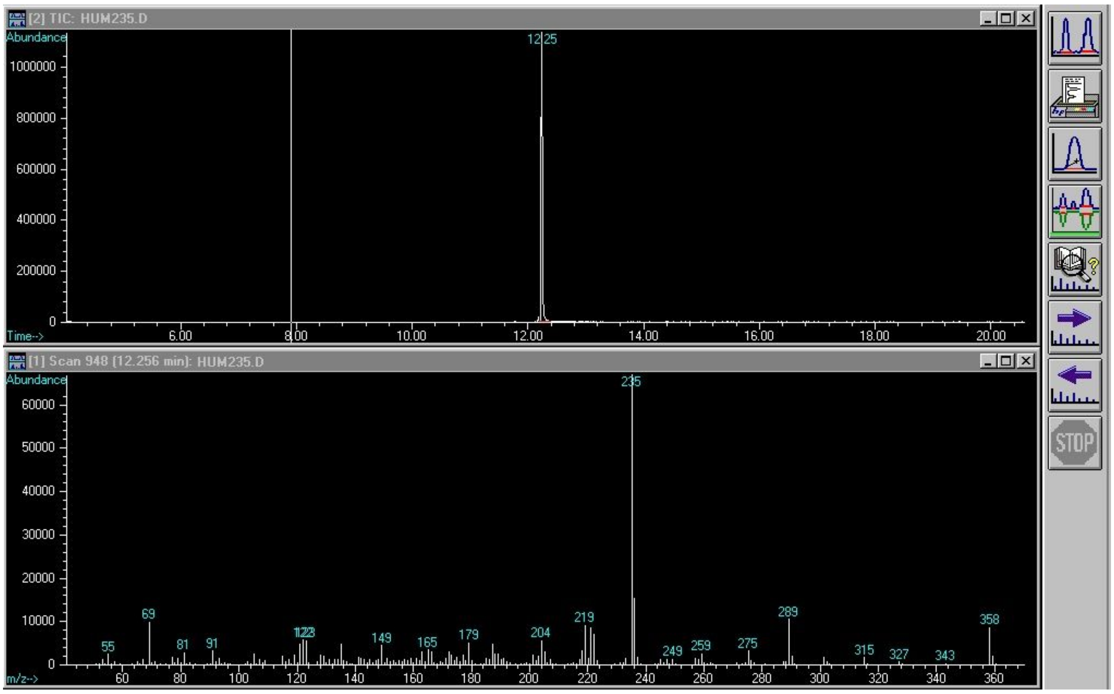

Area Percent Report -- Sorted by Signal

Retention Time	Area	Area %	Ratio %	Type	Width
Total Ion Chromatogram					
12.246	18034182	100.000	100.000	VB	0.026

Figure S20. GCMS chromatogram and purity report for Compound 16
